# Thermodynamic study of the effect of ions on the interaction between dengue virus NS3 helicase and single stranded RNA

**DOI:** 10.1038/s41598-019-46741-4

**Published:** 2019-07-22

**Authors:** Leila A. Cababie, J. Jeremías Incicco, Rodolfo M. González-Lebrero, Ernesto A. Roman, Leopoldo G. Gebhard, Andrea V. Gamarnik, Sergio B. Kaufman

**Affiliations:** 1 Departamento de Química Biológica, Facultad de Farmacia y Bioquímica, Universidad de Buenos Aires and Instituto de Química y Fisicoquímica Biológicas-CONICET, Ciudad Autónoma de Buenos Aires, C1113AAD Argentina; 2 Departamento de Ciencia y Tecnología, Universidad Nacional de Quilmes and CONICET, Bernal, Buenos Aires B1876BXD Argentina; 3 Fundación Instituto Leloir and IIBBA-CONICET, Ciudad Autónoma de Buenos Aires, C1405BWE Argentina

**Keywords:** Thermodynamics, RNA

## Abstract

Dengue virus nonstructural protein 3 (NS3) fulfills multiple essential functions during the viral replication and constitutes a prominent drug target. NS3 is composed by a superfamily-2 RNA helicase domain joined to a serine protease domain. Quantitative fluorescence titrations employing a fluorescein-tagged RNA oligonucleotide were used to investigate the effect of salts on the interaction between NS3 and single stranded RNA (ssRNA). We found a strong dependence of the observed equilibrium binding constant, *K*_obs_, with the salt concentration, decreasing at least 7-fold for a 1-fold increase on cation concentration. As a result of the effective neutralization of ~10 phosphate groups, binding of helicase domain of NS3 to ssRNA is accompanied by the release of 5 or 7 monovalent cations from an oligonucleotide or a polynucleotide, respectively and of 3 divalent cations from the same oligonucleotide. Such estimates are not affected by the type of cation, either monovalent (KCl, NaCl and RbCl) or divalent (MgCl_2_ and CaCl_2_), nor by the presence of the protease domain or the fluorescein label. Combined effect of mono and divalent cations was well described by a simple equilibrium binding model which allows to predict the values of *K*_obs_ at any concentration of cations.

## Introduction

Dengue virus (DENV) is an important human pathogen that belongs to the Flaviviridae family together with other emergent and re-emergent viruses such as Zika, yellow fever, West Nile and hepatitis C viruses^1^. Found in more than one hundred countries around the world, DENV causes over 390 million infections per year^2^ and puts about half of the world population at risk of infection^3,4^.

The Flaviviridae genome is a plus-stranded RNA molecule that is translated into a unique polyprotein which is subsequently processed by viral and host proteinases into 10 mature viral proteins: three structural (capsid, premembrane, and envelope proteins) and seven non-structural proteins (NS1, NS2A, NS2B, NS3, NS4A, NS4B and NS5)^5–7^.

DENV NS3 protein contains 618 amino acids with two well defined domains. At the N-terminal region, amino acid residues 1 to 170, together with part of the NS2B protein, form an active serine protease and at the C-terminal region, amino acid residues 171 to 618 folds into a tri-lobed domain with RNA-helicase, RNA 5’-triphosphatase (RTPase), nucleoside 5’-triphosphatase (NTPase), and RNA annealing activities^8–11^. *In vitro*, these two functional domains appear to work independently of each other. In this regard, it has been previously shown that the recombinant full-length NS3 protein (NS3f) including both the protease and helicase domains, and the NS3 helicase domain alone (NS3h) exhibit similar helicase and NTPase activities^9–12^. Nevertheless, the protease domain has been proposed to modulate the helicase/NTPase activities^13^. To further investigate this possibility, the NS3h and NS3f variants of the NS3 protein were included in this study.

RNA helicases are ubiquitous proteins that intervene in practically all pathways of RNA metabolism^14^. An extended property of these motor proteins is their ability to catalyze the hydrolysis of nucleotides (commonly ATP), which supplies the driving force for directional movement along RNA strands and for unwinding of double stranded RNA tracks.

The tertiary structure of DENV NS3h^13^ is shaped by an arrangement of two RecA-like folds, which includes the distinctive sequence motifs of superfamily 2 helicases^15,16^ and determines the core of many ATP-driven molecular motors^14,17,18^. The cleft between the RecA-like subdomains of NS3h (residues 171 to 481) and its C-terminal subdomain (residues 482 to 618) display numerous basic residues and is wide enough to harbor an ssRNA but not a duplex^13,19^.

Binding of a helicase to a polynucleotide track is the first necessary step of the mechanism of translocation and unwinding activities. Most *in vitro* studies have shown that RNA-helicases bind predominantly to single stranded RNA -and DNA in some cases- with a defined orientation with respect to the 3′-5′ nucleic acid polarity. Binding displays little or no specificity for particular sequences^20,21^ however *Jankowsky & Harris*^22^ reformulated the traditional specific/nonspecific in terms of affinity distribution along sequence space.

Several studies on nonspecific protein-nucleic acid interactions have provided a general understanding of the forces involved in these interactions as well as a theoretical framework to characterize them experimentally^23^. A general feature of this kind of interactions is their strong dependence on the salt concentration and type^21,24^. Such property has been successfully explained as the result of the net release of counterions from the nucleic acid and, in some cases, from the protein upon formation of the protein-nucleic acid complexes. Indeed, the free energy change proceeding from the release of cations from the nucleic acid to the bulk solution is generally recognized as a mayor contribution to the thermodynamic stability of protein-nucleic acid complexes^24–26^. From an experimental perspective the theory developed by Record *et al*. provides a means to determine the net number of ions released from the analysis of the dependence of the equilibrium binding constant for a given protein-nucleic acid interaction on salt concentration. In addition, this theory allows estimating the effective number of ionic pair interactions established between the protein and the nucleic acid phosphate groups. We hypothesize that this analysis provides information that is not only important to complete the thermodynamic characterization of the non-specific binding of NS3h to ssRNA, but also is relevant in the elucidation of the molecular mechanism of the NS3 helicase as a motor protein.

We have shown previously that DENV NS3h is a monomeric protein, binds nonspecifically to ssRNA with equal estimations for minimum and occluded binding site sizes (among 9 and 11 nucleotide residues), and that binding of several NS3h molecules to the same ssRNA track takes place with weak positive cooperativity^27^. In this study, we report an *in vitro* analysis to characterize the effects of concentration and type of monovalent and divalent cations on the equilibrium binding of DENV NS3h to ssRNA using quantitative fluorescence spectroscopy techniques.

**Table 1:** Parameter values of the linear dependence of log*K*_*obs*_ vs. log[M^+^] for the binding of NS3h to F-p-R_10_.

Salt	∂ log*K*_*obs*_/∂log[M^+^]	log*K*_*obs*_ (1M)
KCl	−5.2 ± 0.4	2.4 ± 0.4
NaCl	−5.3 ± 0.4	2.3 ± 0.4
RbCl	−4.7 ± 0.3	2.9 ± 0.3

Values of log*K*_*obs*_ (1M) were obtained from a linear extrapolation of a plot of log*K*_*obs*_ vs log[M^+^]. Experiments were performed in buffer B_K_ for KCl and RbCl and in buffer B_Na_  for NaCl, at 25.0 °C and pH 6.5.

## Materials and Methods

### General reagents

MgCl_2_, KOH, KCl and 3-(N-morpholino)propanesulfonic acid (MOPS) were obtained from Mallinckrodt Baker (ACS reagents). NaOH and HCl were from Carlo Erba. RbCl, CaCl_2_, adenosine 5′-triphosphate (ATP), 3-[(3-Cholamidopropyl) dimethylammonio]-1-propanesulfonate (CHAPS) and ethylenediaminetetraacetic acid (EDTA) were obtained from Sigma Aldrich. RNAse-free ultrapure water (Arium mini Ultrapure Lab Water, Sartorius) was used to prepare all stock solutions and buffers. Concentrations of CaCl_2_, MgCl_2_ and EDTA in stock solutions were determined by volumetric titrations using a Zn^2+^ standard solution and eriochrome black T indicator^28^.

### Protein purification

In the work presented here, we used two variants of the NS3 protein derived from the cDNA of an infectious clone of the DENV serotype 2^29^. These proteins were expressed and purified in the same way that we explained in our previous works^11,12^. The NS3h variant is composed of residues 171 to 618 corresponding to the NTPase/helicase domain of the NS3 protein. The variant NS3f is an ensemble composed of the central region of the NS2B protein (residues 49 to 95) joined via a flexible linker peptide (GGGGSGGGG) to the full length NS3 protein (residues 1 to 618) so as to include both the protease domain and the NTPase/helicase domain. Additionally, the His51 residue corresponding to the catalytic triad of the protease domain was replaced by alanine to prevent auto-proteolytic degradation. Moreover, a hexa-histidine tag derived from the pET-28a plasmid was added to the N-terminal region of each protein construct to allow purification by affinity chromatography. The eluted fractions carrying the protein of interest were dialyzed twice at 4 °C in dialysis buffer (50 mM MOPS-KOH, pH 6.5 at 25 °C, 0.2 mM DTT, 200 mM KCl, 5% w/v glycerol). The protein samples were concentrated to 10 mg/ml using a centrifugal filter unit (Amicon Ultra, Millipore Corp.) and then centrifugated to remove any precipitated material. Supernatants were aliquoted and stored at −70 °C until use. Protein preparations were evaluated by SDS-PAGE and Coomassie staining to determine their homogeneity. Protein concentration was calculated by spectrophotometry according to the Edelhoch method^30^ using the empirical extinction coefficients obtained for NS3f and NS3h in our dialysis buffer (e_280nm,25°C_ = 108.25 mM^−1^∙cm^−1^ and 73.13 mM^−1^∙cm^−1^, respectively)^12^.

### RNA oligo and polynucleotides

Fluorescein-labeled and unlabeled RNA oligonucleotides were purchased from Midland CRC (Texas, USA). These oligonucleotides were purified by HPLC by the manufacturer. The lyophilized oligonucleotides were dissolved in water and their concentrations were calculated by spectrophotometry. The RNA oligonucleotides (both F-p-R_10_ and p-R_10_) have the same sequence 5′-AGUUGAGUUG-3′ and are phosphorylated at their 5′ ends, so both contain 10 phosphate groups. The F-p-R_10_ oligo differs from p-R_10_ in which incorporates a fluorescein moiety covalently attached to the 5′ terminal phosphate group through a phosphodiester bond.

Polyadenylic acid (poly(A), potassium salt, Catalog number P-3001) and polyuridylic acid (poly(U), potassium salt, Catalog number P-3004) were from Midland Certified Reagent Company. Both nucleic acids were dissolved in 10 mM MOPS/KOH buffer (pH 6.5 at 25 °C, 2.0 mM K^+^) and stored at −80 °C. Their concentrations in mononucleotide units were determined spectrophotometrically^31^ employing the following molar extinctions coefficients: 10.3 mM^−1^∙cm^−1^ at 260 nm for poly(A) and 9.2 mM^−1^∙cm^−1^ at 260 nm for poly(U)^32^. According with specification of supplier, an analytical polyacrylamide gel electrophoresis showed nearly all of the polymer of poly(A) to be greater than 250 nucleotides in length and nearly all of the polymer of poly(U) to be less than 250 nucleotides in length.

### Equilibrium fluorescence titrations

Steady-state fluorescence measurements were performed as described in our previous study^27^. Binding of NS3 variants to the fluorescent probeF-p-R_10_ was monitored in a Jasco FP-6500 spectrofluorometer equipped with a Peltier thermostat by following the fluorescence emitted between 510 and 640 nm upon excitation at 495 nm. Emission and excitation slits were set at 3 nm bandwidth and all measurements were done in a 3 mm-pathlength quartz cuvette. A linear correlation between the fluorescence intensity and the F-p-R_10_ concentration was checked in our experimental conditions. Due to the very low absorbance of the samples at the excitation wavelength of 495 nm (less than 0.005 for all the reaction mixtures), the inner filter effects were negligible (less than 1% of the observed signal) and therefore it was not necessary to apply corrections^33^. Fluorescence spectra of different mixtures of NS3 and ssRNA were measured repeatedly until no further change occurred between measurements. Approximately 30 minutes of incubation were enough to achieve constancy to all conditions tested. The mixtures were allowed to stand in reaction tubes (Greiner Bio-One) at 25 °C in a thermomixer prior to recording the fluorescence spectra^27^. In this way, concentrations of all the species present in the reaction mixtures correspond to those of the equilibrium^27^. Relative fluorescence change was defined as:

1 $${\rm{\Delta }}{F}_{rel}=\frac{F-{F}_{0}}{{F}_{0}}$$

where *F* is the fluorescence intensity at a given NS3 protein variant concentration and *F*_0_ denotes the fluorescence intensity of the free F-p-R_10_.

#### Fluorescence titrations of F-p-R_10_ and competition titrations of p-R_10_ with NS3 protein

Titration curves were analyzed using a single-site binding isotherm:

2 $${\rm{\Delta }}{F}_{rel}={\rm{\Delta }}{F}_{max}(\frac{{K}_{obs}[{\rm{NS3}}]}{1+{K}_{obs}[{\rm{NS3}}]})$$

where *K*_*obs*_ is the macroscopic association constant describing the binding of NS3 to the oligonucleotide and *ΔF*_*max*_ is the maximum relative fluorescence increase. Note that Eq. 2 is a function of the concentration of free NS3, which is unknown, and must be solved in terms of the total concentrations of both NS3 and RNA to fit this equation to the experimental titration curves:

3 $${\rm{\Delta }}{F}_{rel}={\rm{\Delta }}{F}_{max}(\tfrac{{K}_{obs}+{[{\rm{NS3}}]}_{total}+{[F \mbox{-} \mathrm{RNA}]}_{total}-\sqrt{{({K}_{obs}+{[{\rm{NS3}}]}_{total}+{[F \mbox{-} \mathrm{RNA}]}_{total})}^{2}-4{[{\rm{NS3}}]}_{total}{[F \mbox{-} \mathrm{RNA}]}_{total}}}{2{[F \mbox{-} \mathrm{RNA}]}_{total}})$$

Competition titration experiments were achieved according to the procedure defined by Jezewska *et al*.^34^ Briefly, if the fluorescent (F-RNA) and non-fluorescent (RNA) macromolecules compete for their binding to the ligand (NS3), mass balance on the ligand should read as:

4 $${[{\rm{NS3}}]}_{total}={[{\rm{NS3}}]}_{free}+{[F \mbox{-} \mathrm{RNA}]}_{total}{\nu }_{f}+{[{\rm{RNA}}]}_{total}{\nu }_{u}$$

where the binding density, ν, for labeled and unlabeled RNA are identified by subscripts f and u, respectively. Regardless of the total concentration of unlabeled macromolecule, a given value of ΔF_*rel*_ would be reached at a unique value of free ligand concentration and thus of ν_u_^34^.

#### Reaction media

Titration assays were performed at 25 °C in buffer B_K_ (25.0 mM MOPS/KOH, 0.50 mM EDTA and 0.50 mM CHAPS) or B_Na_ (25.0 mM MOPS/NaOH, 0.50 mM EDTA and 0.50 mM CHAPS), pH 6.5 (25 °C), containing variable amounts of monovalent and divalent salts (KCl, NaCl or RbCl and MgCl_2_ or CaCl_2_, respectively). As reported in a previous work, CHAPS detergent was added to avoid problems likely due to sticking of the protein to the reaction tubes and/or tips walls^27^. In the range of protein and salt concentrations employed we did not detect the formation of protein aggregates. An interesting observation in this regard is that, while the formation of aggregates is dependent on the total concentration of monovalent salts (data not shown) and they are rapidly formed at KCl concentrations below 20 mM at low concentrations of MgCl_2_^27^, aggregation was not observed in mixtures containing low concentrations (~10 mM) of KCl in the presence of MgCl_2_ concentrations above 10 mM. This observation suggests that the formation of aggregates is prevented by the increase in the ionic strength.

The amount of monovalent cation proceeding from different sources was taken into account to compute total monovalent cation concentrations in the reaction media. The sources of monovalent cation were: the corresponding chloride salt (MCl) solutions, the titration of the MOPS buffer with KOH or NaOH solutions to the desired pH and the stock solutions of EDTA, protein and nucleic acid. The value of Mg-EDTA dissociation constant used to calculate the concentration of free Mg^2+^ in equilibrium was taken from the literature^35^.

#### Competition titrations with homopolyribonucleotides poly(U) and poly(A)

Mixtures of NS3 and the F-p-R_10_ oligonucleotide were titrated with poly(U) and poly(A). Fluorescence measurements were done as described previously but carried out in a 1 cm-pathlength cuvette equipped with a magnetic stirrer. Titrant solutions of polynucleotide contained the same concentrations of NS3 and F-p-R_10_ as the solution in the cuvette, so that corrections for dilution were not required. The initial volume in the cell was 1600 or 1800 μl and aliquots from 20 μl to 300 μl of poly(U) or poly(A)were added. Fluorescence emission spectra (495 nm → 510 to 640 nm) were recorded ~5 to 15 min after the addition of each aliquot.

The binding of NS3 to poly(U) and poly(A) was analysed with McGhee-von Hippel formalism for non-specific interactions of large ligands to a one-dimensional lattice of infinite length^27,36^. The parameters of the binding to F-p-R_10_ were set to those obtained in independent direct titrations of this oligonucleotide.

The mass balance equation for NS3 is:

5 $${[{\rm{NS3}}]}_{total}=[{\rm{NS3}}]+{[F \mbox{-} \mathrm{RNA}]}_{total}{\nu }_{f}+{[\mathrm{poly}({\rm{N}})]}_{total}{\nu }_{pN}$$

where ν_*f*_ is the binding density for the labeled oligonucleotide and ν_*pN*_ is the binding density for poly(U) or poly(A),

6 $${\nu }_{pN}=\frac{[{{\rm{NS3}}}_{pN}]}{{[\mathrm{poly}({\rm{N}})]}_{total}}$$

where [NS3]_*pN*_ is the concentration of NS3 bound to poly(U) or poly(A), and [poly(N)]_total_ is the total concentration of poly(U) or poly(A) in nucleotide residues. According to the formalism of McGhee and von Hippel^36^ the generalized equation for the binding density is^27^:

7 $${\nu }_{pN}=[{\rm{NS3}}]{K}_{pN}(1-m{\nu }_{pN}){[\frac{2{\omega }_{pN}(1-m{\nu }_{pN})}{(2{\omega }_{pN}-1)(1-m{\nu }_{pN})+{\nu }_{pN}+r}]}^{m-1}\,{[\frac{1-(m+1){\nu }_{pN}+r}{2(1-m{\nu }_{pN})}]}^{2}$$

where r is given by

8 $$r=\sqrt{{(1-{\nu }_{pN}-m{\nu }_{pN})}^{2}+4{\omega }_{pN}{\nu }_{pN}(1-m{\nu }_{pN})}$$

K_*pN*_, m and *ω*_*pN*_ denote the intrinsic association constant, the occluded site size and the cooperativity factor, respectively, characterizing the binding of NS3 to the corresponding homopolyribonucleotide. The occluded site size was set to 10 nucleotides according to our previous study^27^.

### Data analysis

Model simulations and nonlinear regression analysis of the data were performed using Copasi version 4.13 (http://www.copasi.org/) and Libreoffice 4.2 spreadsheets (http://www.libreoffice.org/). Figures were edited in QtiPlot version 0.9.8.9 (http://www.qtiplot.com/).

## Results

Previous studies from our laboratory have shown that NS3h is a monomeric protein with a ssRNA binding site size of 9 nucleotides^27^. Those experiments were performed at a fixed set of reaction conditions: pH 7.0, 30 °C, 1.9 mM free Mg^2+^ and 100 mM K^+^, with both cations added in the form of their chloride salts^27^. In the present work, we extend the analysis to characterize the effect of ions on the equilibrium constant for the binding of NS3 to ssRNA. To this aim, we performed fluorescence titration experiments using the signal proceeding from a fluorescein-labeled oligonucleotide (F-p-R_10_, see *Materials and Methods*) for which we have shown that NS3h binds with a 1:1 stoichiometry^27^.

### Effect of monovalent cation concentration and type on the binding of NS3h to RNA oligonucleotide

Fluorescence titrations of the F-p-R_10_ oligonucleotide with the NS3h protein were carried out in buffer B (pH 6.5, 25 °C) in the presence of different concentrations of monovalent salts, KCl, NaCl and RbCl. As described in *Materials and Methods*, buffer B contains a basal amount of about 5 mM of monovalent cations K^+^ (buffer B_K_) or Na^+^ (buffer B_Na_) proceeding from the pH adjustment of the MOPS buffer and EDTA solution with KOH or NaOH, respectively. Additionally, a small amount of K^+^ (about 2 mM) is provided by the dialysis buffer in which the purified NS3h protein was stored, which was compensated with the variation of protein concentration along each titration curve. The rest of the monovalent cation to reach the total given concentration is added in the form of chloride salt.

**Figure 1:**
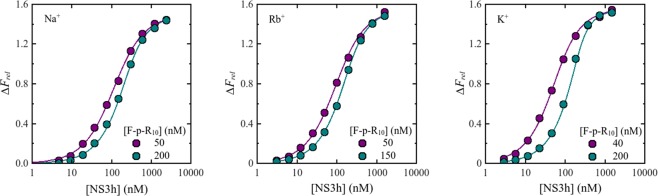
Representative fluorescence titration of F-p-R_10_ with NS3h. Dot symbols represent experimental values and solid lines are simulated titration curves based on Eq. 3 with parameter values obtained by non-linear regression analysis: KCl: *K*_*ob*s_ = 3.6 × 10^7^ M^−1^, *ΔF*_*ma*x_ = 1.6, RbCl: *K*_*obs*_ = 1.5 × 10^7^ M^−1^, *ΔF*_*ma*x_ = 1.6 y NaCl: *K*_*obs*_ = 1.2 × 10^7^ M^−1^, *ΔF*_*ma*x_ = 1.5. Binding of NS3h to F-p-R_10_ was monitored by fluorescence emission from 510 nm to 640 nm upon excitation at 495 nm. Titration experiments were performed in buffer B_K_ or B_Na_ supplemented with 130 mM NaCl, 120 mM RbCl and 105 mM KCl at 25.0 °C and pH 6.5.

Each experiment consisted of titrations of F-p-R_10_ with NS3 were done at two or three oligonucleotide concentrations. Figure 1 shows representative titrations in the presence of K^+^, Na^+^ or Rb^+^. At higher F-p-R_10_ concentration, more NS3 concentration is necessary to get the same value of Δ*F*_*rel*_. This is because at higher RNA concentrations more ligand is necessary to get identical degree of binding.

Consistent with a 1:1 binding stoichiometry, each set of titration curves was well depicted by Eq. 3 with best-fitting values of the observed macroscopic association constant *K*_*obs*_ and the maximum relative fluorescence increase (Δ*F*_*max*_) acquired from non-linear regression analysis of the results (solid lines in Fig. 1).

**Figure 2:**
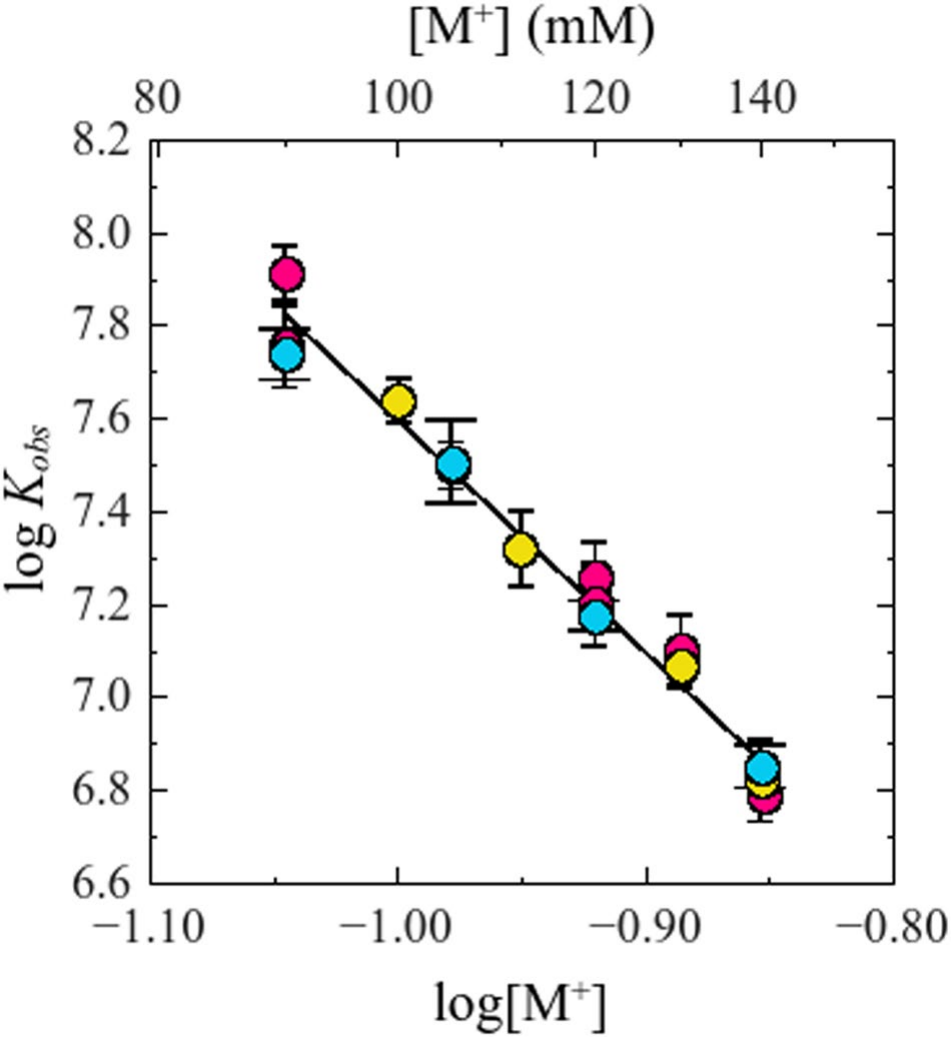
Effect of monovalent cations on *K*_*obs*_ for the interaction NS3h:F-p-R_10_. Experiments were performed with a series of salts differing in cation type: () NaCl, () KCl and () RbCl (plotted as total [M^+^]). Solid line is the representation of Eq. 9 with parameter values obtained from an overall fit by linear least-square analysis: *a* = 5.0 and *b* = 2.6. Experiments were carried out in buffer B_K_ or B_Na_, at 25.0 °C and pH = 6.5.

Logarithm of *K*_*obs*_ is plotted in Fig. 2 as a function of the logarithm of total monovalent cation concentration [M^+^] (Notice that, in order to apply logarithms, the quantities involved must of course be dimensionless. In accordance with usual practice this is implicitly done referring concentrations and equilibrium constant to 1 molar, M, standard states. That is, all logarithms are applied over the numerical values obtained when the quantities are expressed in molar units). It is observed that, within the experimental error, equal values of *K*_*obs*_ were obtained for the same values of [M^+^], regardless of the nature of the added cation, K^+^, Na^+^ or Rb^+^_._ In other words, the effect of monovalent salts on the binding of NS3h to the fluorescein-labeled oligonucleotide shows no specificity for the cation involved.

Additionally, these results show a strong dependence of *K*_*obs*_ on the salt concentration, decreasing about 25-fold for a 1 fold increase on [M^+^], which is a common property of protein-nucleic acid interactions^37^.

In the range of salt concentration tested, 90 to 140 mM of M^+^, the logarithm of *K*_*obs*_ varies linearly with the logarithm of [M^+^] (Fig. 2) and this dependence can be described by the following equation:

9 $$log{K}_{obs}=-\,a\,log[{M}^{+}]+b$$

where clearly −*a* is the slope (∂log*K*_*obs*_/∂log[M^+^])_pH,T_ of the straight line defined by this equation, and *b* is the value of log*K*_*obs*_ at the reference state [M^+^] = 1 M. Linear regression analysis of the results obtained in the presence of K^+^, Na^+^ and Rb^+^ provides the values of *a* and *b* shown in Table 1. These values were not significantly different for the three cations tested and simultaneous linear fitting to all the results provides the values (±standard error) *a* = 5.0 ± 0.2 and *b* = 2.6 ± 0.2.

The linear dependence of log*K*_*obs*_ on log[M^+^] may be interpreted and analyzed by means of the theoretical formalism developed by Record *et al*. to analyze the linkage effects of multiple equilibria with small ligands on the binding between macromolecules^24,38^. This formalism indicates that, in the absence of preferential interactions of ions with the protein and of differential hydration effects, the slope of the dependence of log*K*_*obs*_ on salt concentration, (∂log*K*_*obs*_*/*∂log[M^+^])_pH,T_, is dictated by the net number of cations released from the nucleic acid upon binding of the protein (*a* in the following reaction scheme)^38^. The intercept *b* is identified as the logarithm of the ‘thermodynamic’ equilibrium constant, log*K*_*T*_, of the binding reaction shown below, in which the release of cations is made explicit.

$$NS3+RNA\leftrightarrow RNA-NS3+a{M}^{+}\,{K}_{T}=\frac{[RNA-NS3].{[{M}^{+}]}^{a}}{[NS3][RNA]}$$

According with this interpretation, binding of DENV NS3h to the ssRNA oligonucleotide is accompanied by the release of 5.0 ± 0.2 monovalent cations M^+^. Using the linear fit value of *b*, one obtains *K*_T_ = 392 ± 1 (referred to 1 M standard state for all reactants). Although *K*_T_, unlike *K*_*obs*_, does not depend on [M^+^], still retains possible dependencies on other variables such as pH, buffer composition and temperature.

Notice that our analysis does not take into account potential effects of chloride ions^39^, whose concentration changes together with the concentration of cation. Thus, the meaning attributed to the value of the slope, -*a*, remains in a hypothetical ground and is open to further verification (see below on the discussion about the effect of divalent cations).

Regarding the spectroscopic parameter, *ΔF*_*max*_, we observed variations between experiments. However, it did not appear to be dependent on the salt concentration (Fig. S1 in supplementary information) and take a mean value 2.50 ± 0.05.

### Testing the impact of the fluorescein label: binding of NS3h to the unlabeled RNA oligonucleotide

In order to test whether the fluorescein label contributes to the salt dependence of the observed association constant, we performed competition titration experiments employing the unlabeled counterpart of the F-p-R_10_ oligonucleotide, p-R_10_. This unlabeled oligonucleotide, unlike those used in our previous work^27^, contains the same number of phosphate groups as the labeled one.

F-p-R_10_ at 30 or 50 nM concentration was titrated with NS3h in the presence of different concentrations of the p-R_10_ oligonucleotide, in buffer B_K_ (pH 6.5, 25 °C) at different concentrations of KCl.

**Figure 3:**
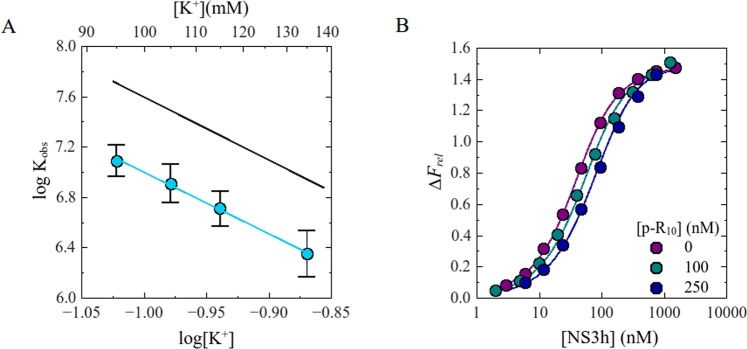
Panel A. Effect of KCl on *K*_*obs*_ for the binding of NS3h to the unlabeled p-R_10_ oligonucleotide. Dot symbols represent log*K*_*obs*_ values for the binding of NS3h to the unlabeled analogue of the F-p-R_10_ oligonucleotide and the blue solid line (−) represents graphically Eq. 9 with parameter values obtained by linear least-square fit: *a* = 4.9 and *b* = 2.1. Black solid line (−) is the representation of the same equation with the corresponding parameters for the binding of NS3h to F-p-R_10_. Experiments were carried out in buffer B_K_ at 25.0 °C and pH 6.5. Panel B. Representative competition titration experiments of F-p-R_10_ and p-R_10_ with NS3h. Dots symbols represent experimental values and solid lines are simulated titration curves based on Eqs 3 and 4 with parameter values obtained by non-linear regression analysis: *K*_*obs*_ = 2.5 × 10^7^ M^−1^ and Δ*F*_*max*_ = 1.8. Binding of NS3h to F-p-R_10_ (20 nM) was monitored by fluorescence emission from 510 nm to 640 nm upon excitation at 495 nm. Titration experiments were performed in buffer B_K_ at 25 °C and pH 6.5 supplemented with 95 mM KCl and the indicated concentration of the p-R_10_ oligonucleotide.

Each set of titration curves (at a given K^+^ concentration) was numerically analyzed employing a competition model in which labeled and unlabeled oligonucleotide compete with a 1:1 stoichiometry for their binding to NS3h (see Materials and Methods and Gebhard *et al*^27^.). Values of *K*_*obs*_ for the unlabeled oligonucleotide was determined by least-square fitting of the model, setting the value of *K*_*obs*_ for the labeled oligonucleotide to the value calculated from Eq. 9 using the parameters *a* and *b* obtained from the results shown in Fig. 2. The results were satisfactorily described by this competition model (Fig. 3).

Values of *K*_*obs*_ for the unlabeled p-R_10_ oligonucleotide were ~4-fold lower than for the F-p-R_10_ oligomer (Fig. 3) which is in agreement with the change in intrinsic affinity found in a previous work (;^27^ see Discussion). log*K*_*obs*_ varied linearly with log[M^+^], with slope −*a* = −4.9 ± 0.2 and ordinate *b* = 2.1 ± 0.2. Thus, binding of NS3h to the unlabeled oligonucleotide is accompanied by a net release of 5 monovalent cations from the nucleic acid, in agreement with the value obtained for the labeled oligonucleotide.

### Effect of monovalent cations on the binding of NS3h to polynucleotides

It has been previously reported that the number of ions released when a charged ligand binds to a polymeric nucleic acid is different from when it binds to short oligomers^40–42^. This difference was attributed to changes in the number of counterions thermodynamically associated per phosphate groups and explained by the contributions of coulombic end effect^42^. In order to test this phenomenon on the NS3h-RNA interaction we determined the association binding constant of NS3h to poly(A) and poly(U) in the presence of different concentrations of monovalent cations.

**Figure 4:**
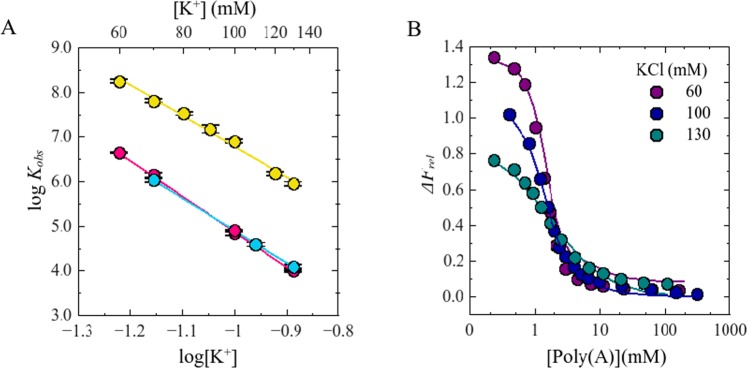
Panel A: Effect of KCl on *K*_*obs*_ for the interactions of NS3 with polynucleotides. Dot symbols represent log*K*_*obs*_ values for the binding of the following pairs: () NS3h:poly(A), () NS3h:poly(U), () NS3f:poly(U). Solid lines are the representations of Eq. 9 with parameters values obtained by linear least-square fits: *a* = 6.0 and *b* = 1.1 for NS3h:poly(**A**), *a* = 7.7 and *b* = −2.8 for NS3h:poly(U) and *a* = 7.2 and *b* = -2.3 for NS3f:poly(U). Panel B: Representative competition titration experiments of NS3h:F-p-R_10_ with poly(A). Dot symbols represent experimental values and the solid lines are representation of Eq. 7 with parameter values obtained by non-linear regression analysis. Binding of NS3h to F-p-R_10_ was monitored by fluorescence emission from 510 nm to 640 nm upon excitation at 495 nm. Titration experiments were carried out in buffer B_K_ at 25.0 °C and pH 6.5.

Mixtures of 50 nM F-p-R_10_ and 0.20 or 0.60 µM NS3h were titrated with poly(A) or poly(U), in buffer B_K_ (pH 6.5, 25 °C) at different concentrations of KCl. The addition of poly(A) or poly(U) caused a decrease in the value of ∆*F*_*rel*_ as expected if both nucleic acids compete with F-p-R_10_ for the binding to NS3h (Fig. 4B, Panel B and Fig. S2 in Supplementary Information). Titration curves were analyzed employing a competition model in which F-p-R_10_ binds to NS3h following a 1:1 stoichiometry (Eq. 3) whereas binding to poly(A) or poly(U) is described using the McGhee-von Hippel formalism for the non-specific interaction of large ligands to a one-dimensional lattice of infinite length^36,43^ (see Materials and Methods). Binding constants for poly(A) and poly(U) at each salt concentration tested were acquired by non-linear regression analysis of the results using this competition model (see Materials and Methods).

In the range of salt concentrations tested, the values of *K*_*obs*_ for poly(U) were ~50–100 times lower than for poly(A) which is in accordance with the results obtained in a previous work^27^. Figure 4 shows that log*K*_*obs*_ for poly(A) or poly(U) decreased linearly with log[K^+^]. Linear regression analysis of the results employing Eq. 9 provided the following best fitting values for *a* and *b*: 6.0 ± 0.2 and 1.1 ± 0.2 for poly(A) and 7.7 ± 0.5 and −2.8 ± 0.6 for poly(U). These results indicate that 6 monovalent cations are released when NS3h binds to poly(A), and 8 monovalent cations are released when it binds to poly(U).

Using the limiting-law predicted by the counterion condensation theory^44,45^, Record *et al*^24,38^. showed that, in the absence of preferential interaction of ions with the protein and differential hydration effects, the slope of the dependence of log*K*_*obs*_ on log[M^+^] does not depend on M^+^ concentration and can be computed as the product of two constant factors:

10 $${(\frac{\partial log{K}_{obs}}{\partial log[{M}^{+}]})}_{pH,T}=-\,a=-\,m^{\prime} \psi $$

where *m*′ is the number of ionic interactions between positive groups on the ligand (NS3h) and negatively charged phosphate group on the nucleic acid (ssRNA), and ψ is the fraction of a monovalent counterion thermodynamically associated per phosphate in the absence of protein and its value is a characteristic property of the nucleic acid^24,45^. In all, the empirical Eq. 9 is reformulated as:

11 $$log{K}_{obs}=-\,m^{\prime} \psi log[{M}^{+}]+log{K}_{T}$$

This equation is of great practical use because by knowing the value of ψ, the estimation of the number of ionic interactions involved in a nucleic acid-protein system is straightforward. In this way, substituting in Eq. 11 the known value of *ψ* for poly(U) (*ψ* = 0.74 ± 0.04)^46,47^ and the value of slope obtained here, we estimate that NS3h establishes 10.4 ± 0.5 ionic interactions with poly(U). This estimate is coincident with both the occluded and minimum binding site sizes estimated for the NS3h protein, between 9 to 11 nucleotides^12,27^. We do not perform the same analysis with the results obtained from poly(A) because in this case the number of ion released does not depend linearly with the valence of oligocation^48^.

### Testing the impact of the NS3 protease domain on the association between full-length NS3 and ssRNA

In this section we extended the study of the effect of monovalent cations on the NS3-ssRNA interaction using a full-length construct of the NS3 protein (NS3f) which includes its N-terminal protease domain (see Materials and Methods).

**Figure 5:**
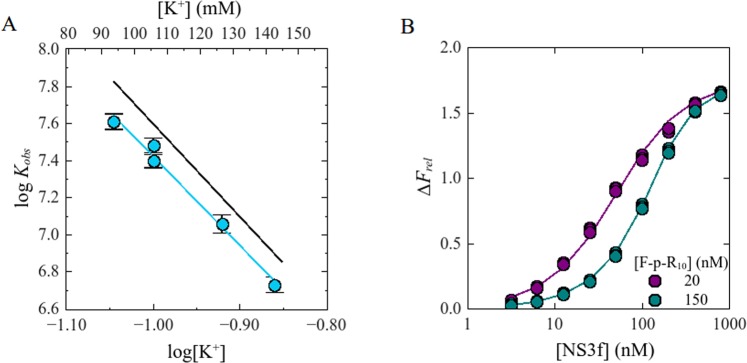
Panel A: Effect of KCl on *K*_*obs*_ for the interaction of NS3f with F-p-R_10_. Dot symbols represent log*K*_*obs*_ values for the binding of full-length construct of NS3 to the oligonucleotide and blue solid line (−) is the representation of Eq. 9 with parameters values obtained by linear least-square fits: *a* = 5.0 and *b* = 2.6. Black solid line (−) is the representation of the same equation with the corresponding parameters for the binding of NS3h to F-p-R_10_. Experiments were carried out in buffer B_K_ at 25.0 °C and pH 6.5. Panel B: Representative fluorescence titration of F-p-R_10_ with NS3f. Dot symbols represent experimental values and solid lines are simulated titration curves based on Eq. 3 with parameter values obtained by non-linear regression analysis: *K*_*obs*_ = 2.5 × 10^7^ M^−1^ and *ΔF*_*max*_ = 1.8. Binding of NS3h to F-p-R_10_ was monitored by fluorescence emission from 510 nm to upon excitation at 495 nm. Titration experiments were performed in buffer B_K_ supplemented with 100 mM KCl at 25 °C and pH 6.5.

We conducted fluorescence titrations of the F-p-R_10_ oligonucleotide with the NS3f protein in buffer B_K_ (pH 6.5, 25 °C) in the presence of different concentrations of monovalent cation K^+^. Titration curves were satisfactorily described by a 1:1 ligand binding model defined by Eq. 3 (Fig. 5B, Panel B) which was fitted to the experimental results to obtain values of *K*_*obs*_ and Δ*F*_*max*_.

In the range of K^+^ concentrations tested, the values of *K*_*obs*_ for NS3f were 1.4 to 1.6 times smaller than for the NS3h (Fig. 5). As with the NS3h construct, log*K*_*obs*_ varied linearly with log[M^+^], with *a* = 4.8 ± 0.3 and *b* = 2.6 ± 0.3 (Eq. 9). Despite the differences in the absolute values of *K*_*obs*_, these linear fit parameters are not significantly different from those obtained for the NS3h construct. According to the theory exposed in the preceding sections, this result indicates that binding of NS3f is accompanied by the release of ~5 monovalent cations from the ssRNA oligonucleotide.

Having characterized the salt effect on the interaction between NS3f and an oligonucleotide (F-p-R_10_), we next examined the effect of monovalent cation concentration on the binding to a polynucleotide (poly(U)). To this end, mixtures of 50 nM F-p-R_10_ and 400 nM NS3f were titrated with poly(U), in buffer B_K_ (pH 6.5, 25 °C) at different concentrations of KCl, and the relative fluorescence increase ∆*F*_*rel*_ was recorded (Fig. S2, Panel A). These results were analyzed as previously described in Material and Methods.

Values of *K*_*obs*_ for the binding of NS3h and NS3f to poly(U) were equal and ~2 orders of magnitude lower than for *F-p-*R_10_. Note that in the range of *K*_*obs*_ values obtained in these experiments, 2 pM^−1^, it is not possible to detect a change of 1.4–1.6 times, such as was observed for the binding of both constructs to *F-p-*R_10._

Once again, log*K*_*obs*_ varied linearly with log[M^+^] (Fig. 4), with *a* = 7.2 ± 0.1 and *b* = -2.3 ± 0.1 (Eq. 9). In accordance with the theory presented above, this result indicates that binding of NS3f is accompanied by the release of ~7 monovalent cations from the poly(U) that is practically equal to the results obtained with the helicase domain NS3h.

### Effect of divalent cations concentration and type on the binding of NS3h to F-p-R_10_ oligonucleotide

Analogously to the experiments presented in the previous sections, we performed fluorescence titrations of the F-p-R_10_ oligonucleotide with the NS3h protein in buffer B_K_ (pH 6.5, 25 °C, 6 mM basal K^+^) in the presence of different concentrations of divalent salts CaCl_2_ and MgCl_2_. Total K^+^ concentration was adjusted to 10 mM with KCl. Given that buffer B_K_ contains 0.50 mM EDTA, the concentration of what we call ‘free divalent cation’ -not bound to EDTA- is not equal to total salt concentration and was instead computed taken into account the equilibrium of metal-EDTA complexation (See Material and Methods).

**Figure 6:**
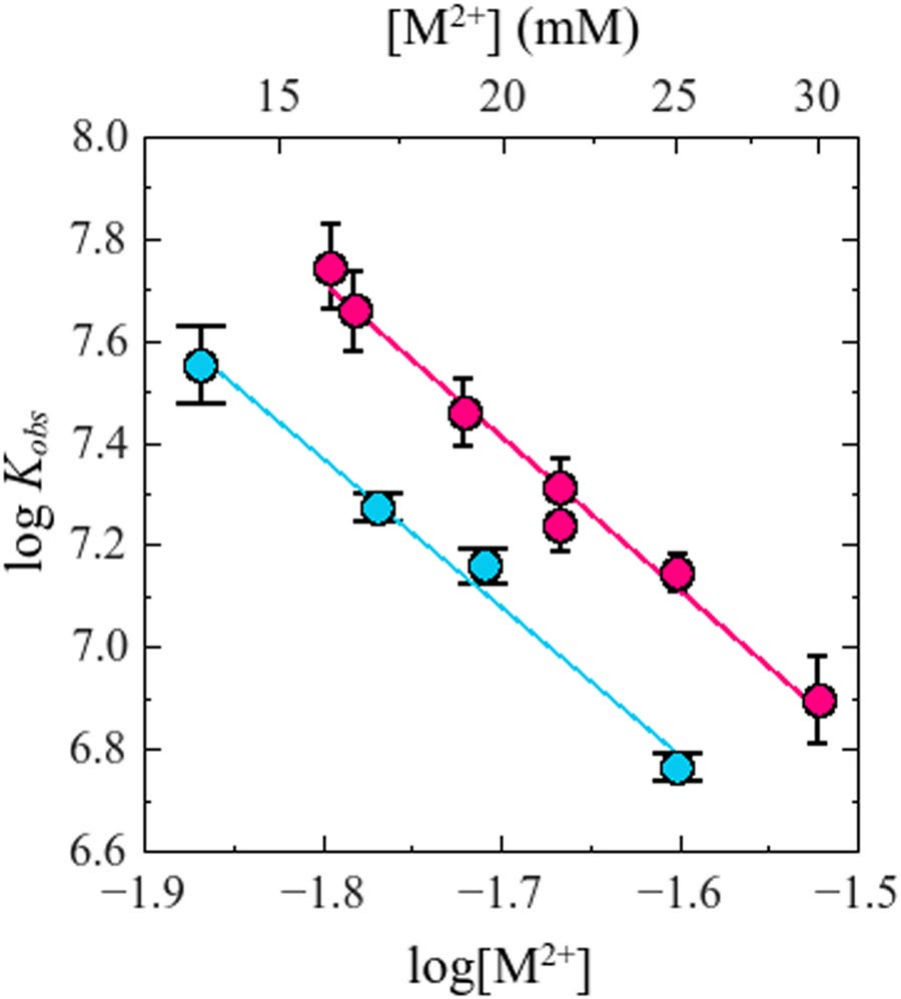
Effect of divalent cations on *K*_*obs*_ for the interaction of NS3h with F-p-R_10_. Experiments were performed with a series of salts differing in cation type: () CaCl_2_ and () MgCl_2_ (plotted as total [M^2+^]). Solid line is the representation of Eq. 9 with parameters values obtained by linear least-square fits: *a* = 3.0, *b* = 2.3 and *a* = 2.9, *b* = 2.1 for the experiments performed with CaCl_2_ and MgCl_2_ respectively. Experiments were carried out in buffer B_K_ containing 10 mM K^+^ at 25.0 °C and pH 6.5.

Titration curves were satisfactorily described by the equations corresponding to a single-site binding model (Eqs 2 and 3). Figure 6 shows the dependence of the logarithm of best-fitting values of *K*_*obs*_ on the logarithm of free divalent cation concentration, log[Mg^2+^] and log[Ca^2+^].

Unlike what was observed for monovalent salts NaCl, KCl and RbCl, values of *K*_*obs*_ were different for the two divalent cations (Fig. 6), being about 2 fold greater in the presence of Ca^2+^ than of Mg^2+^. For both salts tested, log*K*_*obs*_ varied linearly with the logarithm of divalent cation concentration and with practically equal values of slope (−2.9 ± 0.2 for MgCl_2_ and −3.0 ± 0.1 for CaCl_2_).

As will be shown in the following section, these slopes, obtained at 10 mM K^+^, are not expected to be significantly different from the values that would be observed in the absence of monovalent cation. This is because in the range of divalent cation concentrations tested, monovalent cations would be fully displaced from the oligonucleotide (see Discussion). Therefore, under the same assumptions as in the interpretation given above for the effect of monovalent salts, these results indicate that ~3 divalent cations are released upon binding of NS3h to the ssRNA oligonucleotide. Importantly, the ratio between the experimentally observed slopes for divalent and monovalent cations,

12 $$\frac{{(\frac{\partial log{K}_{obs}}{\partial log[{M}^{2+}]})}_{pH,T}}{{(\frac{\partial log{K}_{obs}}{\partial log[{M}^{+}]})}_{pH,T}}=0.59\pm 0.05$$

satisfies the expected relation for a charged ligand-ssRNA interaction in which the concentration of anions (chloride in this case) has no appreciable effects on the association constant *K*_*obs*_, i.e.,

13 $$\frac{-m^{\prime} \sigma }{-m^{\prime} \psi }=\frac{\sigma }{\psi }\simeq 0.60$$

where *ψ* is the same as in Eq. 10 and σ denotes the number of divalent cations bound to the nucleic acid per phosphate residue and its value is a characteristic property of the nucleic acid^47,49^ (see Discussion).

### Combined effects of monovalent and divalent cations

A complete characterization of the effects of monovalent and divalent cations on the interaction between NS3h and ssRNA requires an experimental examination of their combined effects. This is because both cations are counterions of the nucleic acid which compete with each other and with the protein for association to the nucleic acid^24,38,39,49,50^, and therefore their effects on the NS3h-ssRNA binding reaction are necessarily linked.

**Figure 7:**
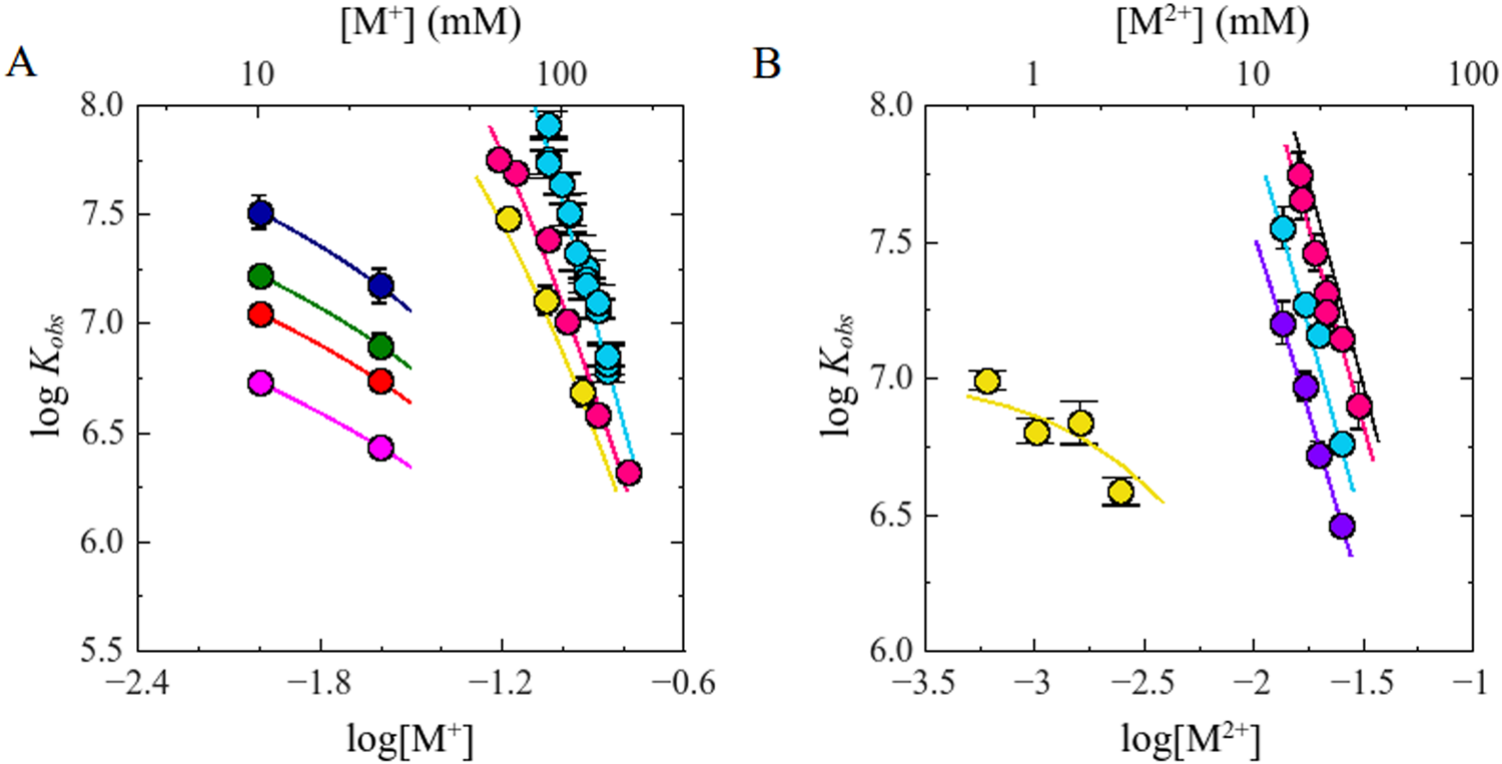
Combined effect of monovalent and divalent cations on *K*_*obs*_ for the interaction of NS3h with F-p-R_10_. Panel A: log*K*_*obs*_ values are shown as a function of M^+^. Dots symbols represent data obtained from experiments performed in the presence of () non MgCl_2_, () 3,() 5, () 13.5, () 17, () 19.5 and () 25 mM MgCl_2_. Solid lines are representations of Eq. 14 with parameter values obtained by non-linear regression analysis: *K*_*T,H*_ = 170 M^−1^ and *K*_*T,Mg*_ = 2.1 M^−1^. Panel B: log*K*_*obs*_ values are shown as a function of M^2+^. Dot symbols (), (), () represent data obtained from experiments performed with MgCl_2_ at 10, 25 and 130 mM KCl respectively. Red dot () indicated experiments carried out in the presence of CaCl_2_ at 10 mM KCl. Solid lines are representations of Eq. 14 with best parameter values obtained by non-linear regression analysis of all the data simultaneously. Black solid line simulates the performance of the model in the absence of M^+^. Titration experiments were performed in buffer B_K_ at 25.0 °C and pH 6.5.

We performed fluorescence titrations of the F-p-R_10_ oligonucleotide with the NS3h protein in buffer B_K_ (pH 6.5, 25 °C) in the presence of different concentrations of K^+^ and Mg^2+^. In Fig. 7, Panel A shows the dependence of the logarithm of best-fitting values of *K*_*obs*_ on log[K^+^] obtained at different magnesium concentrations whereas Panel B shows the dependence upon log[Mg^2+^] at different potassium concentrations. The range of salt concentration tested was set by the technical limits in the determination of *K*_obs_. A limitation is given by the minimum concentration of the fluorescent oligonucleotide, F-p-R_10_, that can be used to obtain a detectable spectroscopic signal with adequate precision. At the other extreme, at high fluorescent oligonucleotide concentrations, loss of linearity or saturation of the spectroscopic signal occur. When increasing NS3 concentration, the difficulties derived from protein aggregation and self-assembly are also present. So that the range of values of *K*_obs_ that we can determine is approximately between 2 × 10^6^ and 8 × 10^7^ M^−1^. As Mg^2+^ concentration is increased, the plots of log*K*_*obs*_ as a function of log[K^+^] becomes increasingly non-linear. The flattening of the plots at low K^+^ concentrations is an expected behavior due to the fact that Mg^2+^ displaces K^+^ from the RNA oligonucleotide and does so more readily at low concentrations of its competing ligand; therefore, as more Mg^2+^ is added, less K^+^ ions are released from the RNA upon binding of the NS3h protein^38,39,47,49^. The observation that the curves tend to converge as K^+^ concentration is increased is explained on the same grounds as the result of competition between both cations.

In order to provide a quantitative and unified description of the simultaneous dependence of *K*_*obs*_ on K^+^ and Mg^2+^ concentration we interpreted the effect of Mg^2+^ as the result of its competitive binding to the nucleic acid which reduces the effective concentration of protein binding sites, such as was done in previous works^39,47,51^. In this view, Mg^2+^ binding to the nucleic acid is assumed to take place through a definite number of binding sites -occupying a definite number of nucleotide residues- and thus it is described by the conventional binding isotherm for the nonspecific interaction of a large ligand to a one-dimensional lattice^52^.

Taking into account that the F-p-R_10_ oligonucleotide contains 10 phosphate groups and assuming that NS3h binding involves the displacement of cations from all of them (*m*′ = 10 from *Gebhard et al*^27^.) and that binding of Mg^2+^ to the oligonucleotide involves a ~3:1 phosphate:Mg^2+^ stoichiometry (see Supplementary Material Text S1), the simultaneous effect of K^+^ and Mg^2+^ concentrations on *K*_*obs*_ can be condensed in the following expression:

14 $${K}_{obs}={K}_{T.}{[{M}^{+}]}^{-a}(\frac{1}{1+{\sum }^{}coe{f}_{0,i}{[{{\rm{Mg}}}^{2+}]}^{i}{K}_{T,{\rm{Mg}}}^{i}{[{M}^{+}]}^{-{a}_{{\rm{Mg}}}i}})$$

where [M^+^] is the total concentration of monovalent cation, *K*_*T*_ and *a* are the same as defined above, *K*_*T*,Mg_ is the intrinsic binding constant for Mg^2+^ ions in the presence of 1 M monovalent cation and *a*_Mg_ denotes the number of monovalent cations released from the nucleic acid upon binding of Mg^2+^. Finally, *coef*_0,*I*_ denotes the combinatorial coefficient that gives the number of possible configurations for a complex with *i* bound Mg^2+^ ions (and without bound protein) deduced by the Epstein’s formalism for the nonspecific interaction of a large ligand to a one-dimensional lattice^52^ (see Supplementary Material Text S1). In the present case, for a 10-mer oligonucleotide and an occluded site size of 3 nucleotide residues, these coefficients take the values 8, 15 and 4 for *i* = 1, 2 and 3, respectively. As a consequence of the assumptions under which it was deduced, this expression only considers the possibility of binding of Mg^2+^ to the free RNA, not bound to the protein, but its extension to more general cases is straightforward.

Parameters appearing in Eq. 14, *K*_*T*_ and *a* were determined from the results shown in the preceding sections, whereas *a*_Mg_ was computed assuming that each Mg^2+^ ion displaces an amount of monovalent cations proceeding from 3 phosphate residues, which for the F-p-R_10_ oligonucleotide corresponds to 1.5 monovalent cations (see Supplementary Material Text S1). Finally, the value of *K*_*T,Mg*_, was estimated by simultaneous least-square fitting of Eq. 14 to all the results shown in Fig. 7.

Eq. 14 provided a good description of the experimental data (solid lines in Fig. 7) with a best-fit value of *K*_*T,Mg*_ = 2.1 ± 0.2. This result suggests that the effect of MgCl_2_ concentration on the interaction between DENV NS3h and ssRNA can be attributed to the binding of the Mg^2+^ to the nucleic acid, without the need of invoking an interaction of Mg^2+^ or Cl^−^ ions with the protein.

## Discussion

The general properties and interpretation of the salt effect on the nonspecific interaction between proteins and nucleic acids were extensively studied and characterized by Record, Lohman & De Haseth^24,37,39,44,45,53^. These studies developed the theoretical framework and the experimental basis to apply it to any particular protein-nucleic acid system providing thermodynamic information of great interest. In the present study we implemented this analysis as a tool to expand the thermodynamic characterization of the interaction between DENV NS3h and ssRNA.

### Effect of monovalent cations

Fluorescence titration experiments were performed with the fluorescein-labeled RNA oligonucleotide F-p-R_10_ to determine the dependence of observed binding constant *K*_*obs*_ on the concentration of monovalent cations K^+^, Na^+^ and Rb^+^ added in the form of their chloride salts. It was observed that the binding affinity of NS3h for the ssRNA oligonucleotide -quantified as *K*_*obs*_- was not dependent on the nature of the added cation (Fig. 2 and Table 1). This observation suggests that preferential cation interactions with RNA do not differ significantly K^+^, Na^+^ and Rb^+^ as was found in previous studies on the interaction of oligolysines with ssRNA^47,54^.

In all cases, log *K*_*obs*_ decreased linearly with log[M^+^] with a slope (∂log*K*_*obs*_/∂log[M^+^])_pH,T_ of −5.0 ± 0.2. As was stated in *Results*, we interpreted this slope as the result of the preferential interaction of cations with the nucleic acid. It should be noted, however, that in the general case the value of (∂log*K*_*obs*_/∂log[M^+^])_pH,T_ is governed by the preferential interaction coefficients of electrolyte ions (added cation M^+^ and its anion Cl^−^) and H_2_0 with nucleic acid, protein and the complex protein-nucleic acid (cf. Eq. 20 in Record 1991^38^).

With respect to the preferential interaction of water molecules, the net release or uptake of water molecules upon protein-nucleic acid binding can give rise to a non-linear dependence of log*K*_*obs*_ on log[M^+^], which was not observed in our results (Fig. 2). However, theory^24^ and experiments^39^ show that the range of salt concentrations used in our work could be not wide and high enough for non-linearity to be observed, mainly because the sensitivity of slope (∂log*K*_*obs*_/∂log[M^+^])_pH,T_ with respect to log[M^+^] increases exponentially with log[M^+^] (see Eq. 20 in Record 1991^38^). Even in such case where nonlinearity cannot be observed, differential hydration could still affect the value of (∂log*K*_*obs*_/∂log[M^+^])_pH,T_ through an additive term, which sign will be negative or positive if water is released or taken up in the binding reaction. In this sense, employing Eq. 2 from Ha *et al*^55^. we calculated that the release or uptake of about 250 water molecules is equivalent to the release of 1 monovalent cation in its contribution to the value of (∂log*K*_*obs*_/∂log[M^+^])_pH,T_ in the range of salt concentration between 90 mM and 140 mM as employed in our experiments. Given the values observed in previous experimental studies of hydration effects on nonspecific protein-nucleic acid interactions (uptake 1.7 ± 0.3^56^; none^57^; 3 ± 52^55^; none^58^), we think it is appropriate to neglect the potential effects of preferential water interaction on the analysis of the results presented in this work.

The potential effect of preferential interactions of anions -added as counterion of the salt- would proceed from differences in the interaction with free protein and protein bound to the nucleic acid^24^. Under conditions in which the degree of anion binding to the protein increases with salt concentration, slope (∂log*K*_*obs*_/∂log[M^+^])_pH,T_ is also expected to increase with salt concentration. For that reason, as in the case of preferential interactions of water, anion effects can be evidenced by loss of linearity in the plot of log *K*_*obs*_ as a function log[M^+^], which was not observed in our results. Here again, linearity does not ensure that the observed value of slope (∂log*K*_*obs*_/∂log[M^+^])_pH,T_ is not affected by anion release. However, the ratio between slope (∂log*K*_*obs*_/∂log[M^2+^])_pH,T_ (obtained with MgCl_2_ and CaCl_2_) and (∂log*K*_*obs*_/∂log[M^+^])_pH,T_ satisfies the expected value for the case in which anion and hydration effects are absent (see Eq. 13 and below). This fact provides support to the interpretation of −(∂log*K*_*obs*_/∂log[M^+^])_pH,T_ as the net number of monovalent cations released in the NS3-RNA binding reaction^24,39^.

With these considerations in mind, our results indicate that binding of DENV NS3h to the F-p-R_10_ is accompanied by the release of ~5 monovalent cations.

### Testing the dependence of the ion release with the nucleic acid length

We determined that binding of NS3h to poly(U) or poly(A) was more sensitive to KCl concentration than binding to the 10-mer oligonucleotide (Fig. 4). In terms of the interpretation of (∂log*K*_*obs*_/∂log[M^+^])_pH,T_ given throughout this work, the results indicate that one and two/three more monovalent cations are released in the interaction of NS3h with poly(A) (*a* = 6.0 ± 0.3) and poly(U) (*a* = 7.7 ± 0.5), respectively, compared to its interaction with the 10-mer oligonucleotide (*a* ~ 5). This is an expected result. In previous experimental and theoretical studies of binding of oligolysines to single-stranded oligonucleotides and polynucleotides, it was found that (∂log*K*_*obs*_/∂log[M^+^])_pH,T_ increases with nucleic acid length until it reaches a maximum value. This effect of nucleic acid length was interpreted as the result of an increase of the extent of counterion binding to longer nucleic acids due to a higher negative charge density within the nucleic acid strand (away from its 3′ and 5′ ends)^41,42,59^.

To distinguish the properties of the nucleic acid and of the protein contained in the observed values of a (*ψ* and *m*′ in Eq. 10, respectively) we employed as a reference the value of ψ for poly(U) obtained by Mascotti and Lohman^46^ from their study of the binding of oligolysines of different valence to the nucleic acid. Comparison of the values of (∂log *K*_*obs*_/∂log[M^+^])_pH,T_ for the F-p-R_10_ oligonucleotide and poly(U) allowed us to determine a value of *ψ* for the 10-mer oligonucleotide of about 0.50 units, 32% smaller than the known value for poly(U) (0.74). This represents a similar change as that observed for the binding of oligolysines to dT oligomers of increasing lengths, were (∂log*K*_*obs*_/∂log[M^+^])_pH,T_ changed from 3.8 for a 10-mer to 6.4 for poly(dT)^45^.

Results shown in Fig. 3 provided us a value of *m’* = 10 ± 1 (see Eq. 10) which, following Record and co-workers’ interpretation^60^, represents the effective charge on the binding surface of the NS3h implicated in the interaction with RNA. The magnitude of *m’* is coincident with that of the minimum and occluded binding site size of NS3h obtained in previous works^12,27^. Although these two interaction parameters are expected to have some kind of relationship, given that both are related to the extension of the nucleic acid binding site on the protein, there is no reason to expect them to be equal. From a structural point of view, crystallographic data^19^ from DENV4 NS3h bound to a 12- or 13-mer RNA oligomer shows a segment of 7 nucleotide residues, with 6 phosphate groups, in the nucleic acid binding site. These structural data indicate that there are 6 positively charged residues (4 Arg and 2 Lys) and 2 negatively charged Asp residues within a radius of 8 Å from the phosphate groups of the ssRNA. The differences between the number of ionic interactions obtain in this work and that reported from crystallographic studies is not unusual. In this sense Record^38^ suggested that “neutralization of nucleic acid phosphates by proteins is in general not accomplished by classical salt bridges (ion pairs)” and Harrison and Aggarwal^61^ concluded that “hydrogen bonds from positively charged side chains to nucleic acid phosphates appear with only modest frequency. Coulombic interactions from less strongly anchored arginines and lysines do appear to be important, however, since in each structure a number of such residues lie near the DNA backbone”.

### Testing the effect of the NS3 protease domain

Previous studies addressed the effect that the protease domain of NS3 exerts on the NTPase and helicase activities^11–13,27^. Dissimilar results were obtained from such studies which did not permit to reach a general conclusion on this issue. In this work we inquired the impact of the protease domain on the equilibrium interaction of DENV NS3 with ssRNA.

Experimental results shown in Fig. 5 indicate that *K*_*obs*_ for the binding of NS3f to F-p-R_10_ are 1.4–1.6 times smaller than that for NS3h while both constructs released ~5 monovalent cations upon binding to the oligonucleotide. This coincidence could be due to the fact that the number of cations released by each construct is limited by the total number of counterions thermodynamically bound to F-p-R_10_. To test this possibility, we determined the salt dependence of the NS3-poly(U) association constant since the length of this nucleic acid does not limit the number of counterions that could be released upon binding of the protein. (Fig. 4). We found that both, NS3h and NS3f, released the same number of monovalent cations. Taken together, our results indicate that the appending of the protease domain does not affect the value of the parameter *m’* which characterizes the interaction of NS3 with ssRNA.

### Effect of divalent cations and combined effect of mono and divalent cations

Application of counterion condensation theory in the analysis of the results shown in Fig. 6 provides a means to test the validity of the assumption that preferential interactions with small anions and water molecules can be neglected. According to this theory, in the absence of anion effects the ratio between the slopes (∂log*K*_*obs*_/∂log[M^2+^])_pH,T_ and (∂log*K*_*obs*_/∂log[M^+^])_pH,T_ should be equal to *σ/ψ*, the ratio of the number of divalent and monovalent cations thermodynamically bound to the nucleic acid per phosphate residue^49^ (Eqs 12 and 13). This relationship with *σ/ψ* is strictly applicable for slopes obtained from experiments performed in the presence of only one kind of small cation, divalent or monovalent. However, the same relationship may hold if the slopes are obtained in a mixture where the concentration of one of the cations -the one kept constant- is sufficiently small (See Supplementary material, Text S2). This was the condition under which we performed the experiments to determine the value of (∂log*K*_*obs*_/∂log[M^2+^])_pH,T_ (where M^2+^ was Mg^2+^ or Ca^2+^), where a basal amount of K^+^ (10 mM) was present along with the divalent salts tested (Fig. 7). In this sense, fitting of an equilibrium thermodynamic model that takes into account the simultaneous effect of K^+^ and Mg^2+^ on *K*_*obs*_, to the set of experimental results obtained in the presence of both cations (Fig. 7) allowed us to evaluate to what extent the limiting value of (∂log*K*_*obs*_/∂log[M^2+^])_pH,T_ would differ from the value obtained in the presence of 10 mM K^+^. According to our analysis (see Supplementary Information, Text S2) the value of (∂log*K*_*obs*_/∂log [M^2+^])_pH,T_ at 10 mM K^+^ obtained in the range of Mg^2+^ and Ca^2+^ employed would be no more than 3% smaller than its limit value, a difference even lower than the experimental uncertainty.

Therefore, we can interpret the coincidence between the experimental value of the ratio (∂log*K*_*obs*_/∂log[M^2+^])_pH,T_ / (∂log*K*_*obs*_/∂log[M^+^])_pH,T_ (~0.58) and the expected value of *σ/ψ* (~0.60)^49^ as evidence that, if there are preferential interactions of anions and water molecules, they have a negligible effect on the dependence of *K*_*obs*_ on salt concentration.

As was mentioned above, we studied the combined effect of mono and divalent cations on *K*_*obs*_. A simple model fitted to the results provided a means to predict the value of *K*_*obs*_ in any concentration of mono and divalent cations (see the model in Supplementary Information, Text S1). Importantly, the model allowed us to explain the effect of both cations solely as the results of the preferential interaction of the cations with the RNA, without the need to invoke interactions of cations and/or anions with the protein. Additionally, this working model indicates that the difference in *K*_*obs*_ values observed in the experiments performed in the presence of Ca^2+^ or Mg^2+^ (Fig. 6) can be explained by a difference in the binding affinity of these cations for RNA phosphate groups (See Supplementary Information, Text S2).

## Supplementary information

Supplementary Material

## References

[1] Gubler DJ (2002). Epidemic dengue/dengue hemorrhagic fever as a public health, social and economic problem in the 21st century. Trends Microbiol.

[2] Bhatt S (2013). The global distribution and burden of dengue. Nature.

[3] WHO. *Dengue: Guidelines for diagnosis, treatment, prevention, and control*. (World Health Organization, Geneva, 2009). 23762963

[4] Weaver SC, Vasilakis N (2009). Molecular evolution of dengue viruses: contributions of phylogenetics to understanding the history and epidemiology of the preeminent arboviral disease. Infect Genet Evol.

[5] Chambers TJ, Hahn CS, Galler R, Rice CM (1990). Flavivirus genome organization, expression, and replication. Annu Rev Microbiol.

[6] Perera R, Kuhn RJ (2008). Structural proteomics of dengue virus. Curr Opin Microbiol.

[7] Bollati M (2010). Structure and functionality in flavivirus NS-proteins: Perspectives for drug design. Antiviral Res.

[8] Cui T (1998). Recombinant dengue virus type 1 NS3 protein exhibits specific viral RNA binding and NTPase activity regulated by the NS5 protein. Virology.

[9] Li H, Clum S, You S, Ebner KE, Padmanabhan R (1999). The serine protease and RNA-stimulated nucleoside triphosphatase and RNA helicase functional domains of dengue virus type 2 NS3 converge within a region of 20 amino acids. J. Virol..

[10] Bartelma G, Padmanabhan R (2002). Expression, purification, and characterization of the RNA 5’-triphosphatase activity of dengue virus type 2 nonstructural protein 3. Virology.

[11] Gebhard LG, Kaufman SB, Gamarnik AV (2012). Novel ATP-independent RNA annealing activity of the dengue virus NS3 helicase. PLoS ONE.

[12] Incicco JJ, Gebhard LG, González-Lebrero RM, Gamarnik AV, Kaufman SB (2013). Steady-state NTPase activity of dengue virus NS3: Number of catalytic sites, nucleotide specificity and activation by ssRNA. PLoS One.

[13] Xu T (2005). Structure of the dengue virus helicase/nucleoside triphosphatase catalytic domain at a resolution of 2.4 Å. J Virol.

[14] Jankowsky E, Fairman ME (2007). RNA helicases - one fold for many functions. Curr Opin Struct Biol.

[15] Gorbalenya AE, Koonin EV, Donchenko AP, Blinov VM (1989). Two related superfamilies of putative helicases involved in replication, recombination, repair and expression of DNA and RNA genomes. Nucleic Acids Res.

[16] Cordin O, Banroques J, Tanner N, Linder P (2006). The DEAD-box protein family of RNA helicases. Gene.

[17] Wolfe AR, Meehan T (1992). Use of binding site neighbor-effect parameters to evaluate the interactions between adjacent ligands on a linear lattice: Effects on ligand-lattice association. J Mol Biol.

[18] Liao JC (2011). Mechanical transduction mechanisms of RecA-like molecular motors. J Biomol Struct Dyn.

[19] Luo D (2008). Insights into RNA unwinding and ATP hydrolysis by the flavivirus NS3 protein. EMBO J.

[20] Tanner NK, Linder P (2001). DExD/H box RNA helicases: from generic motors to specific dissociation functions. Mol Cell.

[21] von Hippel PHF (2007). “simple” DNA-protein interactions to the macromolecular machines of gene expression. Annu Rev Biophys Biomol Struct.

[22] Jankowsky E, Harris ME (2015). Specificity and nonspecificity in RNA-protein interactions. Nat Rev Mol Cell Biol.

[23] Anderson CF, Record MT (1995). Salt-nucleic acid interactions. Annu Rev Phys Chem.

[24] Record MT, Anderson CF, Lohman TM (1978). Thermodynamic analysis of ion effects on the binding and conformational equilibria of proteins and nucleic acids: the roles of ion association or release, screening, and ion effects on water activity. Q Rev Biophys.

[25] Lohman TM, De Haseth PL, Record MT (1978). Analysis of ion concentration effects on the kinetics of protein-nucleic acid interactions: Application to lac repressor-operator interactions. Biophys Chem.

[26] Overman LB, Bujalowski W, Lohman TM (1988). Equilibrium binding of Escherichia coli single-strand binding protein to single-stranded nucleic acids in the (SSB) 65 binding mode. Cation and anion effects and polynucleotide specificity. Biochemistry.

[27] Gebhard LG (2014). Monomeric nature of dengue virus NS3 helicase and thermodynamic analysis of the interaction with single-stranded RNA. Nucleic Acids Res.

[28] Kolthoff, I. M., Sandell, E. B., Meehan, E. J. & Bruckenstein, S. *Quantitative Chemical Analysis* (ed. Harris, D.) (Macmillan, 1969).

[29] Kinney RM (1997). Construction of infectious cDNA clones for dengue 2 virus: strain 16681 and its attenuated vaccine derivative, strain PDK-53. Virology.

[30] Pace CN, Vajdos F, Fee L, Grimsley G, Gray T (1995). How to measure and predict the molar absorption coefficient of a protein. Protein Sci.

[31] Cavaluzzi MJ, Borer PN, Revised UV (2004). extinction coefficients for nucleoside-5’-monophosphates and unpaired DNA and RNA. Nucleic Acids Res.

[32] Kowalczykowski SC, Lonberg N, Newport JW, Von Hippel PH (1981). Interactions of bacteriophage T4-coded gene 32 protein with nucleic acids: I. Characterization of the binding interactions. J Mol Biol.

[33] Lohman TM, Mascotti DP (1992). Nonspecific ligand-DNA equilibrium binding parameters determined by fluorescence methods. Methods Enzymol.

[34] Jezewska MJ, Bujalowski W (1996). A general method of analysis of ligand binding to competing macromolecules using the spectroscopic signal originating from a reference macromolecule. Application to Escherichia coli replicative helicase DnaB protein-nucleic acid interactions. Biochemistry.

[35] Kolthoff, I. M. *et al*. *Análisis Químico Cuantitativo* (ed. Harris, D.) (Macmillan, 1979).

[36] McGhee JD, von Hippel PH (1974). Theoretical aspects of DNA-protein interactions: Co-operative and non-co-operative binding of large ligands to a one-dimensional homogeneous lattice. J Mol Biol.

[37] Record MT, Lohman TM, De Haseth P (1976). Ion effects on ligand-nucleic acid interactions. J Mol Biol.

[38] Record MT, Ha JH, Fisher MA (1991). Analysis of equilibrium and kinetic measurements to determine thermodynamic origins of stability and specificity and mechanism of formation of site-specific complexes between proteins and helical DNA. Methods Enzymol.

[39] Record MT, De Haseth PL, Lohman TM (1977). Interpretation of monovalent and divalent cation effects on the lac repressor-operator interaction. Biochemistry.

[40] Zhang W, Bond JP, Anderson CF, Lohman TM, Record MT (1996). Large electrostatic differences in the binding thermodynamics of a cationic peptide to oligomeric and polymeric DNA. PNAS.

[41] Zhang W (1999). The importance of Coulombic end effects: experimental characterization of the effects of oligonucleotide flanking charges on the strength and salt dependence of oligocation (L8+) binding to single-stranded DNA oligomers. Biophys J.

[42] Ballin JD, Shkel IA, Record MT (2004). Interactions of the KWK6 cationic peptide with short nucleic acid oligomers: demonstration of large Coulombic end effects on binding at 0.1-0.2 M salt. Nucleic Acids Res.

[43] Bujalowski W, Lohman TM, Anderson CF (1989). On the cooperative binding of large ligands to a one-dimensional homogeneous lattice: The generalized three-state lattice model. Biopolymers.

[44] Manning GS (1969). Limiting laws and counterion condensation in polyelectrolyte solutions I. Colligative properties. J Chem Phys.

[45] Manning GS (1978). The molecular theory of polyelectrolyte solutions with applications to the electrostatic properties of polynucleotides. Q Rev Biophys.

[46] Mascotti DP, Lohman TM (1990). Thermodynamic extent of counterion release upon binding oligolysines to single-stranded nucleic acids. PNAS.

[47] Mascotti DP, Lohman TM (1992). Thermodynamics of single-stranded RNA binding to oligolysines containing tryptophan. Biochemistry.

[48] Mascotti DP, Lohman TM (1993). Thermodynamics of single-stranded RNA and DNA interactions with oligolysines containing tryptophan. Effects of base composition. Biochemistry.

[49] Lohman TM, Mascotti DP (1992). Thermodynamics of ligand-nucleic acid interactions. Methods Enzymol.

[50] De Haseth PL, Lohman TM, Record MT (1977). Nonspecific interaction of lac repressor with DNA: an association reaction driven by counterion release. Biochemistry.

[51] Lohman TM, De Haseth PL, Record MT (1980). Pentalysine-deoxyribonucleic acid interactions: a model for the general effects of ion concentrations on the interactions of proteins with nucleic acids. Biochemistry.

[52] Epstein IR (1978). Cooperative and non-cooperative binding of large ligands to a finite one-dimensional lattice. A model for ligand oligonucleotide interaction. Biophys Chem.

[53] Lohman TM (1986). Kinetics of protein-nucleic acid interactions: use of salt effects to probe mechanisms of interaction. CRC Crit Rev Biochem.

[54] Mascotti DP, Lohman TM (1997). Thermodynamics of oligoarginines binding to RNA and DNA. Biochemistry.

[55] Ha JH, Capp MW, Hohenwalter MD, Baskerville M, Record MT (1992). Thermodynamic stoichiometries of participation of water, cations and anions in specific and non-specific binding of lac repressor to DNA. J Mol Biol.

[56] Galletto R, Jezewska MJ, Bujalowski W (2004). Multistep sequential mechanism of Escherichia coli helicase PriA protein-ssDNA interactions. Kinetics and energetics of the active ssDNA-searching site of the enzyme. Biochemistry.

[57] Garner M, Rau D (1995). Water release associated with specific binding of gal repressor. EMBO J.

[58] Sidorova NY, Rau DC (1996). Differences in water release for the binding of EcoRI to specific and nonspecific DNA sequences. PNAS.

[59] Olmsted MC, Anderson CF, Record MT (1989). Monte Carlo description of oligoelectrolyte properties of DNA oligomers: range of the end effect and the approach of molecular and thermodynamic properties to the polyelectrolyte limits. PNAS.

[60] Shkel IA, Ballin JD, Record MT (2006). Interactions of cationic ligands and proteins with small nucleic acids: analytic treatment of the large coulombic end effect on binding free energy as a function of salt concentration. Biochemistry.

[61] Harrison SC, Aggarwal AK (1990). DNA recognition by proteins with the helix-turn-helix motif. Annu Rev Biochem.

