# Supplementary material for: Thermodynamic study of the effect of ions on the interaction between dengue virus NS3 helicase and single stranded RNA
Source: Sci Rep. 2019 Jul 22;9:10569. doi: 10.1038/s41598-019-46741-4 (PMC6646317; doi:10.1038/s41598-019-46741-4)
Supplement: Supplementary file 1 — Supplementary Material [file 41598_2019_46741_MOESM1_ESM.docx]

# **Thermodynamic study of the effect of ions on the interaction between dengue virus NS3 helicase and single stranded RNA**

Leila A. Cababie^1^, J. Jeremías Incicco^1^, Rodolfo M. González-Lebrero^1^, Ernesto A. Roman^1^, Leopoldo G. Gebhard^2^, Andrea V. Gamarnik^2^, Sergio B. Kaufman^1,^*

^1^ Instituto de Química y Fisicoquímica Biológicas and Departamento de Química Biológica, Facultad de Farmacia y Bioquímica, Universidad de Buenos Aires, Ciudad Autónoma de Buenos Aires, C1113AAD, Argentina.

^2^ Fundación Instituto Leloir-Consejo Nacional de Investigaciones Científicas y Técnicas, Ciudad Autónoma de Buenos Aires, C1405BWE, Argentina.

* To whom correspondence should be addressed. Tel: +54 11 49648289 ext. 131; Fax: +54 11 49625457; Email: [sbkauf@qb.ffyb.uba.ar](mailto:sbkauf@qb.ffyb.uba.ar).

## Supplementary Material


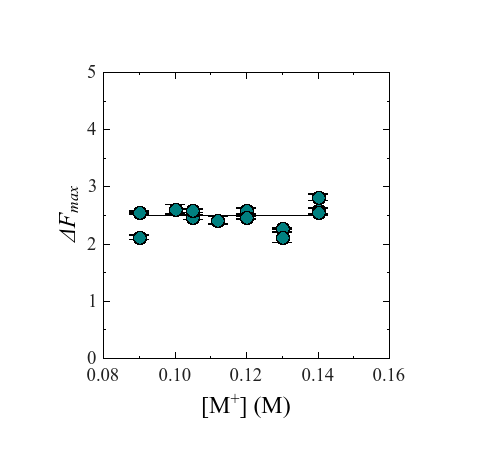


Figure S1. Effect of monovalent cation on *ΔF_max_* for the interaction NS3h/F-p-R_10_. Dot symbols represent *ΔF_max_* values estimated according to equation 3 and solid line represent the mean value = 2.50.


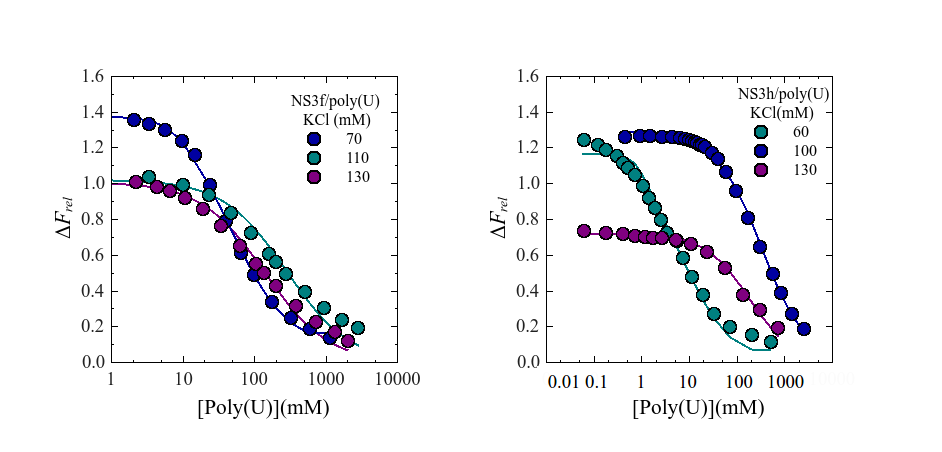


Figure S2. Representative competition titrations of NS3 / F-p-R_10_ with poly(U). Dot symbols represent experimental values and the solid lines are representations of equation 7 with parameter values obtained by non-linear regression analysis. Binding of NS3 to F-p-R_10_ was followed by recording the fluorescence signal after irradiating the sample at 495 nm. Titration experiments were carried out in buffer B_K_ at 25.0 ºC and pH 6.5.

### Text S1: Binding model used in the analysis of the combined effect of monovalent and divalent cations.

In this section, we describe the binding model employed to interpret the results obtained in the presence of both monovalent (K^+^) and divalent (Mg^2+^) cations. The approach, which is equivalent to that applied in previous works [55], is developed in the two following steps:

1) binding of divalent cations to the nucleic acid is explicitly described by a binding polynomial (obtained by the combinatorial Epstein’s formalism; see below) and it is assumed that the protein can bind only to stretches of nucleic acid free of divalent cations,

2) the effect of monovalent cation concentration on the effective association constant for the binding of the protein and divalent cation to the nucleic acid (*K_obs_*), resulting from cation release from the nucleic acid, is then introduced.

*1.A. General formulation of divalent cation effects in terms of binding polynomials assuming binding to the nucleic acid only.*

Taking into consideration that only one NS3h molecule can bind to the RNA oligonucleotide and that divalent cations (M^2+^) can bind to both free nucleic acid and to the protein-nucleic acid complex (but not to the protein), we can formulate the total concentration of free RNA (not bound to the protein) and of NS3h-RNA complex in terms of the following binding polynomials or grand canonical partition functions:

$\left[ \text{RNA} \right] = \left[ \text{R}^{0} \right] + \sum\left[ \text{R}\left( \text{M}^{\text{2+}} \right)_{i} \right] = \left[ \text{R}^{0} \right].P_{R,M\text{2+}}$ (S1)

$\left[ \text{NS3h-RNA} \right] = \left[ \text{NR}^{0} \right] + \sum\left[ \text{NR}\left( \text{M}^{\text{2+}} \right)_{j} \right] = \left[ \text{NR}^{0} \right].P_{NR,M\text{2+}}$ (S2)

where [R^0^] and [NR^0^] denotes RNA and NS3h-RNA complex with no divalent cations bound, respectively; *n_R_* and *n_NR_* are the maximum stoichiometries of M^2+^ binding; and *P_R,M2+_* and *P_NR,M2+_* are the binding polynomials of M^2+^ binding to free RNA and to the NS3h-RNA complex, respectively. Assuming that the sequential binding of divalent cations to the nucleic acid takes place without M^2+^-M^2+^ or NS3h-M^2+^ cooperative interactions and that they bind with the same association constant to all available positions along the nucleic acid, *P_R,M2+_* and *P_NR,M2+_* can be expressed as follows:

$P_{R,M\text{2+}} = 1 + \sum{coef}_{0,i}\left[ \text{M}^{\text{2+}} \right]^{i}K_{obs,M\text{2+}}^{i}$ (S3)

$P_{NR,M\text{2+}} = 1 + \sum\left( \frac{{coef}_{N,j}}{{coef}_{N,0}} \right)\left[ \text{M}^{\text{2+}} \right]^{i}K_{obs,M\text{2+}}^{i}$ (S4)

where coefficients *coef*_0,_*_i_* and *coef_N,j_* denote the combinatorial coefficients that give the number of possible binding configurations for a RNA-M^2+^ complex with *i* bound M^2+^ ions (R(M^2+^)_i_) and for a NS3h-RNA complex with *j* bound M^2+^ ions (NR(M^2+^)_j_), deduced by the Epstein’s formalism for the nonspecific and noncooperative interaction of a large ligand to a one-dimensional lattice (see below). The equilibrium constant *K_obs,M2+_* is the intrinsic association equilibrium constant, which governs the binding of M^2+^ ions to the nucleic acid. It is assumed to be independent of the position along the nucleic acid (nonspecifity assumption) and, as we will make explicit later, it is a function of monovalent cation concentration. Expressed in this form, the binding polynomials contain within *K_obs,M2+_* the activity coefficient for M^2+^.

The observed association equilibrium constant for the binding of NS3h to RNA can therefore be expressed as:

$K_{obs} = \frac{\left[ \text{NS3h-RNA} \right]}{\left[ \text{NS3h} \right].\left[ \text{RNA} \right]} = \frac{\left[ \text{NR}^{0} \right]}{\left[ \text{NS3h} \right].\left[ \text{R}^{0} \right]}.\frac{P_{NR,M\text{2+}}}{P_{R,M\text{2+}}} = K_{obs}^{0}\left[ \frac{1 + \sum\left( \frac{{coef}_{N,j}}{{coef}_{N,0}} \right)\left[ \text{M}^{\text{2+}} \right]^{j}K_{obs,M\text{2+}}^{j}}{1 + \sum{coef}_{0,i}\left[ \text{M}^{\text{2+}} \right]^{i}K_{obs,M\text{2+}}^{i}} \right]$ (S5)

where *K^0^_obs_* is the NS3h-RNA equilibrium constant in the absence of divalent cation M^2+^ at a given concentration of monovalent cation. It is instructive to consider the expression for the effect of M^2+^ concentration on *K_obs_*. Assuming that anion effects proceeding from preferential interactions with the protein are negligible and neglecting nonideality terms (which would be expressed as the effect of M^2+^ concentration on activity coefficients; cf. Record *et al.* 1978 (ref)), we obtain:

$\left( \frac{\partial logK_{obs}}{\partial log\left[ \text{M}^{\text{2+}} \right]} \right)_{pH,T,\left[ M\text{+} \right]} = \left( \frac{\partial logP_{NR,M\text{2+}}}{\partial log\left[ \text{M}^{\text{2+}} \right]} \right)-\left( \frac{\partial logP_{R,M\text{2+}}}{\partial log\left[ \text{M}^{\text{2+}} \right]} \right)$ (S6)

The last terms can be identified as the total binding density of M^2+^ on the NS3h-RNA complex and on the RNA. The difference therefore gives the net number of M^2+^ cations which are *taken up* upon binding of the protein, such that a negative value would indicate a net *release* of M^2+^ cations upon formation of the NS3h-RNA complex. Additionally, although it may result evident it is worth noting that in general this quantity would be a function of M^2+^ concentration. Its constancy may be expected in special cases, such as for preferential interaction effects resulting from counterion condensation to nucleic acids in the presence of only one cation species.

*1. B. Combinatorial coefficients for the binding of divalent cations to the nucleic acid.*

As mentioned, coefficients *coef*_0,_*_i_* and *coef_N,j_* give the number of possible binding configurations for a RNA-M^2+^ complex with *i* bound M^2+^ ions (R(M^2+^)_i_) and for a NS3h-RNA complex with *j* bound M^2+^ ions (NR(M^2+^)_j_), deduced by the Epstein’s formalism for the *nonspecific* and *noncooperative* interaction of a large ligand to a one-dimensional lattice [55]. Their general form is given by the following expressions:

${coef}_{0,i} = \left\{ \begin{matrix} \text{if} L-i.n_{M\text{2+}}\geq0 & , & \frac{\left( L-i.n_{M\text{2+}}+i \right)!}{\left( L-i.n_{M\text{2+}} \right)!i!} \\ & & \\ \text{if} L-i.n_{M\text{2+}}<0 & , & 0 \end{matrix} \right\}$ (S7)

${coef}_{N,j} = \left\{ \begin{matrix} \text{if} L-n_{NS3}-j.n_{M\text{2+}}\geq0 & , & \frac{\left( L-n_{NS3}-j.n_{M\text{2+}}+j+1 \right)!}{\left( L-n_{NS3}-j.n_{M\text{2+}} \right)!j!} \\ & & \\ \text{if} L-n_{NS3}-j.n_{M\text{2+}}<0 & , & 0 \end{matrix} \right\}$ (S8)

where *L* is the nucleic acid length in nucleotide residues (containing phosphate), *n_M2+_* is the effective binding site size of M^2+^ cations and *n_NS3_* is the minimum binding site size of NS3h. It is emphasized that equation S8 was deduced for 1:1 binding stoichiometry between NS3h and RNA (which is the case for the 10-mer oligonucleotide) and assuming that M^2+^ cations are excluded from the region covered by the minimum binding site of the protein.

In applying these equations to the interaction of DENV NS3h with the F-p-R_10_ oligonucleotide we used *L* = 10 and *n_NS3_* = 10 [26]. Therefore, Equation S8 must satisfy *coef_N,0_* = 1 and *coef_N,j_* = 0 for *j* > 0 and thus *P_NR,M2+_* = 1. That is, according to this implementation of the model, there are not available binding sites for M^2+^ in the NS3h-RNA complex.

On the other hand, the effective binding site size of Mg^2+^ (the M^2+^ divalent cation used in the results shown in Figure 7 was estimated not from its net charge but from the maximum Mg^2+^: phosphate stoichiometry predicted by the estimated value for σ_FpR10_ of Equation 13 in the main text. Its value was estimated as follows:

- Firstly, the effective value *ψ* for the F-p-R_10_ oligonucleotide can be computed from the net number of monovalent cations released from the oligonucleotide upon binding of NS3h, *a* = (5.0 ± 0.2), and the value of *m’* determined from the results obtained with poly(U), *m’ =* (10.4 ± 0.5). Therefore, *ψ*_F-p-R10_ = *a*/*m’* = (0.48 ± 0.03) ≈ 0.5.

- Secondly, assuming that the ratio between σ_FpR10_ and ψ_F-p-R10_ satisfies the value obtained by Lohman and coworkers [52] for the binding of oligolysines to poly(U), we can compute σ_FpR10_ as *σ*_FpR10_ = 0.6. *ψ*_F-p-R10_ ≈ 0.3. This value means that, at most, 1 Mg^2+^ cation may bind for each 3 phosphate groups on the oligonucleotide.

Thus, we employed *n_M2+_* = 3. Inserting this value in the expression for *coef_0,i_* given in Equation S7, with *L* = 10, the following values are obtained: 8, 15, 4 and 0 for *i* = {1, 2, 3 and ≥ 4}.

*2.A. Combined effect of monovalent and divalent cation concentration on K_obs_*

The effect of monovalent cation concentration is introduced as a preferential interaction effect proceeding from the release of M^+^ cations from the nucleic acid upon binding of the protein or the divalent cation. That is,

$K_{obs}^{0} = K_{T}.\left[ \text{M}^{\text{+}} \right]^{-a}$ (S9)

$K_{obs,M\text{2+}} = K_{T,M\text{2+}}.\left[ \text{M}^{\text{+}} \right]^{-a_{M\text{2+}}}$ (S10)

where *K_T_* and *K_T,M2+_* are the “thermodynamic” association equilibrium constants, computed for 1 M monovalent cation concentration, for the protein in the absence of M^2+^ and for M^2+^ in the absence of protein, respectively. Coefficients *a* and *a_M2+_* are the number of monovalent cations released upon binding of the NS3h protein and the M^2+^ cation to the RNA oligonucleotide. The value of *a* (~ 5) was obtained from the results shown in Figure 2, whereas the value of *a_M2+_* was computed as *a_M2+_* = ψ_F-p-R10_. *n_M2+_* = 1.5.

Finally, inserting Equations S7 through S10 in Equation S5 we obtain Equation 14 of the main text, shown here for a generic divalent metal cation M^2+^:

$K_{obs} = K_{T.}\left[ \text{M}^{\text{+}} \right]^{-a}\left( \frac{1}{1 + \sum{coef}_{0,i}\left[ \text{M}^{2\text{+}} \right]^{i}K_{T,\text{M2+}}^{i}\left[ \text{M}^{\text{+}} \right]^{-a_{\text{M2+}}i}} \right)$ (S11)

The expression for *K_obs_* in Equation S11 allows us to obtain direct evaluation of the salt dependence of (∂log*K_obs_*/∂log[M^2+^])_pH,T,[M+]_ (as shown more generally in Equation S6):

$\begin{matrix} \left( \frac{\partial logK_{obs}}{\partial log\left[ \text{M}^{\text{2+}} \right]} \right)_{pH,T,\left[ M\text{+} \right]} & = & - \left( \frac{\sum i.{coef}_{0,i}\left[ \text{M}^{2\text{+}} \right]^{i}K_{T,\text{M2+}}^{i}\left[ \text{M}^{\text{+}} \right]^{-a_{\text{M2+}}i}}{1 + \sum{coef}_{0,i}\left[ \text{M}^{2\text{+}} \right]^{i}K_{T,\text{M2+}}^{i}\left[ \text{M}^{\text{+}} \right]^{-a_{\text{M2+}}i}} \right) \end{matrix}$ (S12)

and of (∂logK_obs_/∂log[M^+^])_pH, T, [M2+]_. This later quantity, which -under the assumptions stated and discussed in the main text- provides the net number of monovalent cations released from the nucleic acid upon binding of the protein would be given by:

$\begin{matrix} \left( \frac{\partial logK_{obs}}{\partial log\left[ \text{M}^{\text{+}} \right]} \right)_{pH,T,\left[ M\text{2+} \right]} & = & \left( \frac{\partial logK_{obs}^{0}}{\partial log\left[ \text{M}^{\text{+}} \right]} \right)_{pH,T,\left[ M\text{2+} \right]} - \left( \frac{\partial logP_{R,M\text{2+}}}{\partial log\left[ \text{M}^{\text{+}} \right]} \right)_{pH,T,\left[ M\text{2+} \right]} \\ & & \\ & = & a - a_{M\text{2+}}.\left( \frac{\partial logP_{R,M\text{2+}}}{\partial log\left[ \text{M}^{\text{2+}} \right]} \right)_{pH,T,\left[ M\text{+} \right]} \\ & & \\ & = & a - a_{M\text{2+}}.\left( \frac{\sum i.{coef}_{0,i}\left[ \text{M}^{2\text{+}} \right]^{i}K_{T,\text{M2+}}^{i}\left[ \text{M}^{\text{+}} \right]^{-a_{\text{M2+}}i}}{1 + \sum{coef}_{0,i}\left[ \text{M}^{2\text{+}} \right]^{i}K_{T,\text{M2+}}^{i}\left[ \text{M}^{\text{+}} \right]^{-a_{\text{M2+}}i}} \right) \end{matrix}$ (S13)

The first equality, S12, is directly obtained from Equation S6 considering *P_NR,M2+_* = 1, whereas the second line is obtained through application of the chain rule in the second term. It may be given the following interpretation: the number of monovalent cations released upon binding of the protein in the presence of the divalent cation, (∂logK_obs_/∂log[M^+^])_pH, T, [M2+]_, is equal to the number released in the absence of divalent cation, *a*, minus the number of monovalent cations already released by M^2+^ cations, which is given by *a_M2+_* times the number of M^2+^ bound per RNA molecule.

### Text S2: Interpretation of the results obtained with Ca^2+^ and Mg^2+^ in the presence of 10 mM KCl.

Backed by the model described in the preceding sections, we estimate here to what extent the presence of 10 mM K^+^ would be affecting the value of slopes (∂logK_obs_ /∂log[M^2+^])_pH,T_ obtained from the results shown in Figure 7.

As a reference, in the limit of low [M^+^] values, Equation S12 approximates to:

$\begin{matrix} \left( \frac{\partial logK_{obs}}{\partial log\left[ \text{M}^{\text{2+}} \right]} \right)_{pH,T,\left[ M\text{+} \right]\to0} & = & -\lim_{\left[ M\text{+} \right]\to0} \left( \frac{\sum i.{coef}_{0,i}\left[ \text{M}^{2\text{+}} \right]^{i}K_{T,\text{M2+}}^{i}\left[ \text{M}^{\text{+}} \right]^{-a_{\text{M2+}}i}}{1 + \sum{coef}_{0,i}\left[ \text{M}^{2\text{+}} \right]^{i}K_{T,\text{M2+}}^{i}\left[ \text{M}^{\text{+}} \right]^{-a_{\text{M2+}}i}} \right) \\ & & \\ & = & -\lim_{\left[ M\text{+} \right]\to0} \left( \frac{\sum i.{coef}_{0,i}\left[ \text{M}^{2\text{+}} \right]^{i}K_{T,\text{M2+}}^{i}\left[ \text{M}^{\text{+}} \right]^{a_{\text{M2+}}\left( 3-i \right)}}{\left[ \text{M}^{\text{+}} \right]^{a_{\text{M2+}}3} + \sum{coef}_{0,i}\left[ \text{M}^{2\text{+}} \right]^{i}K_{T,\text{M2+}}^{i}\left[ \text{M}^{\text{+}} \right]^{a_{\text{M2+}}\left( 3-i \right)}} \right) \\ & & \\ & = & 3 \end{matrix}$ (S14)

in the limit where only the terms with higher powers of [M^2+^] persist.

Using the best fitting values of *K_T_*, *K_T,Mg_* and *K_T,Ca_*, we computed the predicted values for (∂logK_obs_/∂log[Mg^2+^])_pH, T_ and (∂logK_obs_ /∂log[Ca^2+^])_pH, T_ at 10 mM K^+^. The values obtained, -2.91 for Mg^2+^ and -2.93 for Ca^2+^, differ in less than 3% with respect to the limit value of 3 predicted by the model.

Another aspect of the results that can be accounted for by the model is the difference in the NS3h-RNA association equilibrium constant (*K_obs_*) observed in the presence of Ca^2+^ and Mg^2+^. We will show here that, according to the model, the difference can be attributed to a difference in the affinity of these two cations for the RNA oligonucleotide.

Using Equation S11 the ratio between *K_obs_* obtained in the presence of Ca^2+^ and *K_obs_* obtained at the same concentration of Mg^2+^ is:

$\left( \frac{K_{obs}^{with{Ca}^{\text{2+}}}}{K_{obs}^{with{Mg}^{\text{2+}}}} \right) = \left( \frac{1 + \sum{coef}_{0,i}\left[ \text{Mg}^{2\text{+}} \right]^{i}K_{T,Mg}^{i}\left[ \text{M}^{\text{+}} \right]^{-a_{\text{M2+}}i}}{1 + \sum{coef}_{0,i}\left[ \text{Ca}^{2\text{+}} \right]^{i}K_{T,Ca}^{i}\left[ \text{M}^{\text{+}} \right]^{-a_{\text{M2+}}i}} \right)$ (S15)

As we noted above, at the concentrations employed in the experiments shown in Figure 7, the quotient is approximately equal to the quotient between the higher powers in divalent cation concentration. Thus, at the same concentration of divalent cations, we have:

$\left( \frac{K_{obs}^{with{Ca}^{\text{2+}}}}{K_{obs}^{with{Mg}^{\text{2+}}}} \right) \approx\left( \frac{K_{T,Mg}}{K_{T,Ca}} \right)^{3}$ (S16)

or equivalently,

$logK_{obs}^{with{Ca}^{\text{2+}}}-logK_{obs}^{with{Mg}^{\text{2+}}} \approx3 \left( logK_{T,Mg}-logK_{T,Ca} \right)$ (S16b)

On this basis, we let the value of *K_T,Ca_*, to be fitted to the results along with *K_T_* and *K_T,Mg_*, assuming that all other interaction parameters of Ca^2+^ were equal to those of Mg^2+^. The values obtained, *K_T,Ca_*  = (1.61 ± 0.02) M^-3^ and *K_T,Mg_*  = (2.16 ± 0.02) M^-3^, account for a difference of log*K_obs_* values of 0.39 units, approximately equal to the observed difference of 0.38 units in the results shown in Figure 7 and S8.
